# Vasohibin 1 selectively regulates secondary sprouting and lymphangiogenesis in the zebrafish trunk

**DOI:** 10.1242/dev.194993

**Published:** 2021-02-19

**Authors:** Marta Bastos de Oliveira, Katja Meier, Simone Jung, Eireen Bartels-Klein, Baptiste Coxam, Ilse Geudens, Anna Szymborska, Renae Skoczylas, Ines Fechner, Katarzyna Koltowska, Holger Gerhardt

**Affiliations:** 1 Integrative Vascular Biology Laboratory, Max-Delbrück Center for Molecular Medicine in the Helmholtz Association (MDC), Robert-Rössle-Strasse 10, Berlin 13125, Germany; 2 DZHK (German Center for Cardiovascular Research), Partner site, Potsdamer Str. 58, 10785 Berlin, Germany; 3 Department of Immunology, Genetics and Pathology, Uppsala University, 752 37 Uppsala, Sweden; 4 Vascular Patterning Laboratory, Center for Cancer Biology, VIB, Leuven B-3000, Belgium; 5 Vascular Patterning Laboratory, Center for Cancer Biology, Department of Oncology, KU Leuven, Leuven B-3000, Belgium; 6 Berlin Institute of Health (BIH), Anna-Louisa-Karsch-Straβe 2, 10178 Berlin, Germany

**Keywords:** Lymphangiogenesis, Vash1, Microtubules, Tubulin detyrosination, Sprouting angiogenesis

## Abstract

Previous studies have shown that Vasohibin 1 (Vash1) is stimulated by VEGFs in endothelial cells and that its overexpression interferes with angiogenesis *in vivo*. Recently, Vash1 was found to mediate tubulin detyrosination, a post-translational modification that is implicated in many cell functions, such as cell division. Here, we used the zebrafish embryo to investigate the cellular and subcellular mechanisms of Vash1 on endothelial microtubules during formation of the trunk vasculature. We show that microtubules within venous-derived secondary sprouts are strongly and selectively detyrosinated in comparison with other endothelial cells, and that this difference is lost upon *vash1* knockdown. Vash1 depletion in zebrafish specifically affected secondary sprouting from the posterior cardinal vein, increasing endothelial cell divisions and cell number in the sprouts. We show that altering secondary sprout numbers and structure upon *Vash1* depletion leads to defective lymphatic vessel formation and ectopic lymphatic progenitor specification in the zebrafish trunk.

## Abstract (precis)

**Summary:** Vasohibin 1-mediated detyrosination of endothelial microtubules is selectively required for the control of venous secondary sprouting and subsequent formation of functional lymphatics in the zebrafish trunk.

## INTRODUCTION

Blood vessel formation and patterning is essential for tissue growth and homeostasis in vertebrate development and physiology. Endothelial cells (EC) arising from the lateral plate mesoderm in early embryonic development initially coalesce at the midline to form the first arterial and venous structures. Subsequent sprouting angiogenesis and remodelling expands and shapes the vascular tree throughout the developing embryo (Hogan and Schulte-Merker, 2017; Potente et al., 2011). Complex morphogenic and cell differentiation processes orchestrate formation of arteries, veins and capillaries, as well as of the lymphatic vascular system.

The trunk vasculature in zebrafish embryos has served both angiogenesis and lymphangiogenesis research as a powerful model to observe, manipulate and mechanistically understand relevant cellular processes and their molecular control (Gore et al., 2016; Hogan and Schulte-Merker, 2017). Following the initial assembly and lumen formation of the dorsal aorta (DA) and posterior cardinal vein (PCV), the intersegmental vessels (ISV) form by sprouting. This first wave of sprouting emerges from the DA at 22 h post fertilization (hpf), with bilateral sprouts along the somite boundaries forming the first vascular loops (Isogai et al., 2003; Kohli et al., 2013). A second wave of sprouting emerges at 34 hpf and consists of venous and lymphatic sprouts (Koltowska et al., 2015; Nicenboim et al., 2015; Yaniv et al., 2006). The venous sprouts remodel half of the ISVs into veins (Geudens et al., 2010; Yaniv et al., 2006). The lymphatic precursor cells are marked by the transcription factor Prox1 in the PCV, before a cell division that results in a differential Prox1 distribution in the daughter cells, influencing their specification and behaviour. Cells retaining high Prox1 levels contribute to sprouts that will form parachordal lymphangioblasts (PLs) at the midline. The PLs subsequently migrate and branch to shape the mature lymphatic system of the zebrafish trunk including the thoracic duct (TD) and the dorsal longitudinal lymphatic vessel (DLLV) (Küchler et al., 2006; Yaniv et al., 2006). Although primary and secondary sprouts appear morphologically similar (Isogai et al., 2003), with acto-myosin cellular protrusions, they are driven by distinct growth factors and downstream signalling cascades. In particular, arterial sprouting involves Vegfa, Kdr and Plcγ, whereas venous/lymphatic sprouting is controlled by Vegfc, Flt4, Ccbe, Adamts3 and Adamts14 (Hogan and Schulte-Merker, 2017; Wang et al., 2020).

Recent reports have highlighted the importance of actin dynamics in EC during morphogenesis (Gebala et al., 2016; Lamalice et al., 2007; Phng et al., 2015). However microtubules, although crucially involved in cell division (Belmont et al., 1990), polarity (Siegrist and Doe, 2007) and vesicle transport mechanisms (Vale, 2003), have received little attention in vascular biology research. This is despite their relevance for tumour angiogenesis and vessel maintenance, as microtubule-targeting drugs used for cancer therapy are anti-angiogenic and/or stimulate the collapse of tumour vasculature (Hayot et al., 2002; Kruczynski et al., 2006; Schwartz, 2009; Shi et al., 2016; Tozer et al., 2002). The mechanisms behind the selectivity of this phenomenon are not understood. Microtubules are unbranched cytoskeleton polymers made of α- and β-tubulin heterodimers that assemble in a directional manner to shape a polarised hollow tube. α-Tubulin carries a tyrosine residue that can be removed post-translationally by the carboxypeptidase Vasohibin 1 (Vash1) (Aillaud et al., 2017; Nieuwenhuis et al., 2017), in a reaction referred to as tubulin detyrosination. Tubulin detyrosination is crucial for neuronal development (Erck et al., 2005), cell division (Barisic et al., 2015; Liao et al., 2019) and influences vesicle transport (Dunn et al., 2008). Interestingly, *VASH1* was previously identified as a VEGFA-inducible gene *in vitro* (Abe and Sato, 2001) and is expressed in the developing endothelium *in vivo* (Hosaka et al., 2009; Shibuya et al., 2006; Watanabe et al., 2004). Viral overexpression as well as VASH1 protein administration experiments *in vitro* and *in vivo* show that VASH1 inhibits angiogenesis and reduces vessel diameter (Hosaka et al., 2009; Kern et al., 2009). Conversely, *Vash1* knockout mice exhibited more vascularised tumours (Hosaka et al., 2009) and increased angiogenesis in the ear skin injury model (Kimura et al., 2009).

Although these studies demonstrated that VASH1 is necessary for controlling angiogenesis in a tumour and injury onset *in vivo*, they predated the discovery of VASH1 as a microtubule-modifying enzyme. Thus, the role of microtubule detyrosination and VASH1 in particular in the context of *in vivo* angiogenesis remains unknown.

Here, we use the zebrafish trunk vasculature to characterise microtubule detyrosination and the effects of *vash1* knockdown (KD) on secondary sprouting and lymphatic development. Using high-resolution live imaging, combined with immunofluorescence and gene expression analysis, we find that endothelial tubulin is predominantly detyrosinated in secondary sprouts. *vash1* KD causes morphogenic defects in secondary but not primary sprouts, impeding proper lymphatic development. Mechanistically, *vash1* loss-of-function leads to increased EC division within secondary sprouts associated with signs of ectopic lymphatic progenitor specification and ultimately defective lymphatic network formation. We propose that Vash1-controlled endothelial tubulin detyrosination supports differential endothelial specification during cell division and sprout elongation to achieve venous and lymphatic vessel formation in the zebrafish trunk.

## RESULTS

### *vash1* is highly expressed in zebrafish EC *in vivo*

**Fig. 1.**
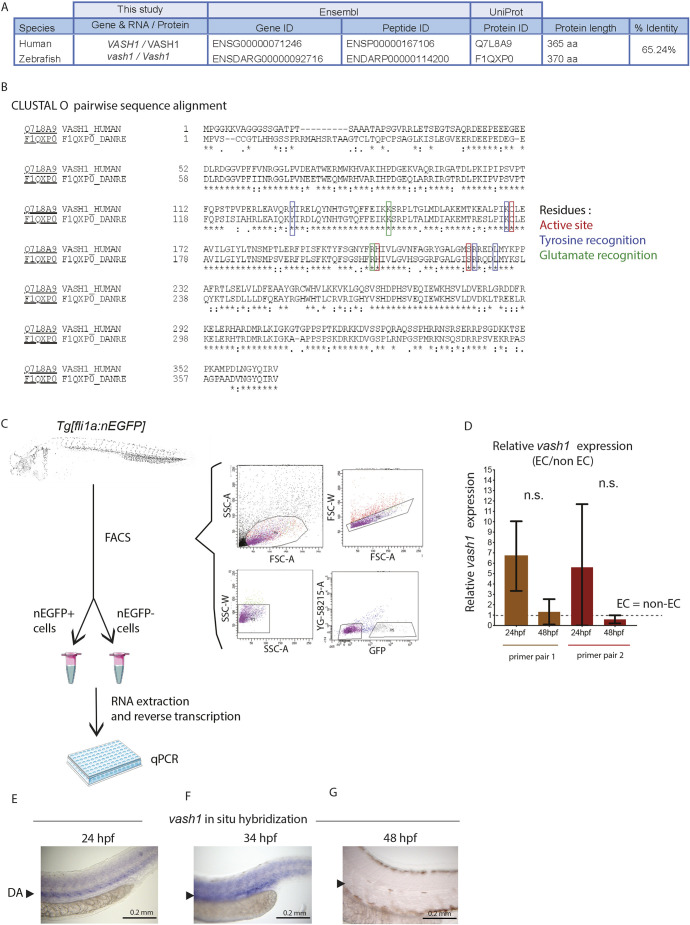
***vash1* is highly expressed in zebrafish endothelium.** (A,B) Amino acid sequence alignment using CLUSTAL O (Uniprot) of human and zebrafish Vash1 showing 65.24% sequence similarity (A), including in the indicated residues necessary for tubulin detyrosination (B). (C,D) Analysis of *vash1* expression in zebrafish embryos at 24 and 48 hpf. FACS sorting and gating strategy for isolation of nEGFP+ and nEGFP− cells from *Tg[fli1a:nEGFP]*^y7^ embryos, their RNA extraction and reverse transcription leading to qPCR (C). *vash1* expression, analysed by two independent primer pairs, shows a tendency towards being enriched in the endothelium of zebrafish embryos at 24 hpf (D). Data normalised to housekeeping genes. Data are mean±s.d. Each data point is an average of three technical replicates per sample, and each experiment has three biological replicates per developmental stage. n.s., not significantly different (Mann–Whitney test). (E-G) *In situ* hybridization of *vash1* in the zebrafish embryo trunk at 24 hpf (E), 34 hpf (F) and 48 hpf (G). Arrowhead indicates where the dorsal aorta (DA) locates in the embryos. Pictures are representative of three replicated experiments. *n*=40 embryos for each developmental stage.

To study the role of Vash1-driven microtubule detyrosination in angiogenesis in zebrafish, we first investigated the conservation of zebrafish and human variants of Vash1 (Fig. 1A,B) with CLUSTAL O (Uniprot). The analysis predicted 65.24% similarity between zebrafish and human Vash1 (Fig. 1A), including the previously identified residues necessary for tubulin tyrosine and glutamate recognition, and the catalytic active site Cys-His-Ser triad (Adamopoulos et al., 2019; Nieuwenhuis et al., 2017; Sanchez-Pulido and Ponting, 2016) (Fig. 1B). RT-qPCR on RNA extracted from EC sorted from *Tg[fli1a:nEGFP]^y7^* embryos at 24 and 48 hpf showed dynamic expression of *vash1* during zebrafish development. During the sprouting phase (24 hpf), *vash1* expression was 5-7 times higher in EC than in non-EC, decreasing at 48 hpf (Fig. 1C,D). Although these differences were not statistically significant, they were independently confirmed with a second primer set. In addition, *in situ* hybridization on fixed wild-type embryos at 24, 34 and 48 hpf showed a variable expression of *vash1* in the trunk (Fig. 1E-G). *vash1* mRNA expression is detectable in the DA and other peri-vascular tissues in the trunk at 24 and 34 hpf (Fig. 1E,F), but not at 48 hpf (Fig. 1G).

In summary, *vash1* is expressed in the zebrafish endothelium and the amino acids responsible for the carboxypeptidase function are conserved in zebrafish.

### Vash1 carboxypeptidase function is conserved in zebrafish

**Fig. 2.**
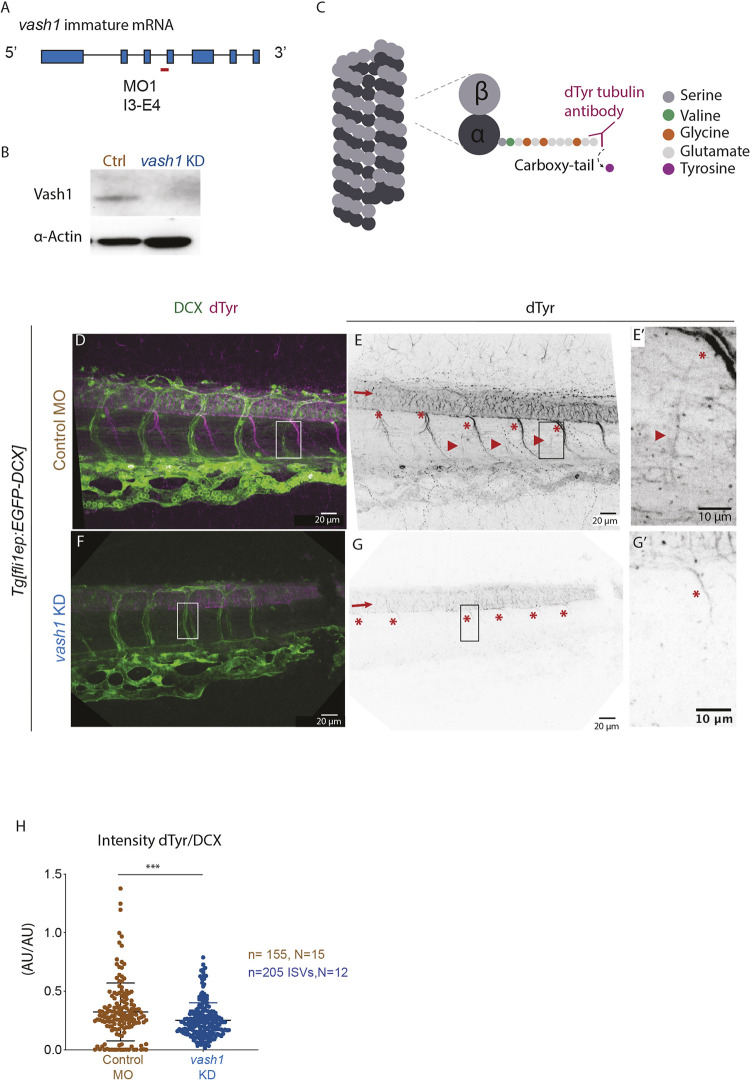
**Endothelial microtubules are detyrosinated by Vash1 in zebrafish.** (A,B) Knockdown (KD) strategy using a morpholino (MO) targeting the intron3-exon4 (I3-E4) (A) efficiently decreases Vash1 protein levels as detected by western blot (B). 48 hpf embryo lysate was used, three replicates were performed. (C) Mechanism of tyrosine cleavage from *α*-tubulin carboxy-terminus by Vash1, resulting in detyrosinated tubulin (dTyr). Detyrosination exposes a glutamate residue accessible by a custom-made antibody (Liao et al., 2019). (D-G′) Immunostainings of 48 hpf *Tg[fli1ep:EGFP-DCX]* embryos detect detyrosinated microtubules (referred to as dTyr; D-G) and GFP-labelled microtubules (referred as DCX; D,F). G and G′ show immunostaining of dTyr upon *vash1* KD, compared with the control MO injected sibling embryos (E,E′). Arrows (E,G) indicate neural tube, with typically detyrosinated microtubules (E), reduced upon *vash1* KD (G). Arrowheads indicate endothelial detyrosinated microtubules, only present in control embryos (E,E′). Asterisks (E,G) indicate motoneurons exhibiting high dTyr signal in control embryos (E,E′) and decreased dTyr signal in *vash1* KD embryos (G,G′). Pictures are representative of three biological replicates. (H) Quantification of ratios between dTyr and DCX intensity signals of each ISV in control and *vash1* KD groups. AU, arbitrary units. Each data point is one ISV, *n*=155 ISVs from 15 embryos (control) and *n*=205 ISVs from 12 embryos (*vash1* KD), from three replicates. Data are mean±s.d. ***P<0.0002 (Mann–Whitney test). Pictures are representative of three replicated experiments.

A splice-site interfering morpholino (MO) targeting the intron3-exon4 boundary of nascent *vash1* RNA (Fig. 2A) efficiently decreased the expression of Vash1 in zebrafish embryos (Fig. 2B). Injections of MO against *vash1* and control MO were performed in the *Tg[fli1ep:EGFP-DCX]* zebrafish line, selectively labelling endothelial microtubules by expression of GFP-tagged microtubule-binding protein doublecortin (Phng et al., 2015). Tubulin detyrosination was assessed by antibody staining against the glutamate residue of α-tubulin that becomes available after the tyrosine residue is removed by Vash1 (Fig. 2C). Microtubules detected by this antibody are referred to as microtubules with detyrosinated tubulin (dTyr). Whereas control embryos exhibited a high intensity array of dTyr in the neural tube, centrosomes, motoneurons (Fig. 2D,E) and EC (Fig. 2E′, arrowhead), *vash1* KD embryos showed strongly diminished labelling of these structures (Fig. 2F,G). In particular, endothelial labelling was absent following *vash1* KD (Fig. 2G′). Quantification of the ratio between dTyr tubulin and DCX (endothelial microtubules) signal intensities (Fig. 2H) confirmed that detyrosination of endothelial microtubules is significantly reduced in *vash1* KD ISVs (dTyr/DCX=0.23±0.15; mean±s.d.) in comparison with control ISVs (dTyr/DCX=0.32±0.24). Thus, Vash1 detyrosinates tubulin in zebrafish tissues including the endothelium.

### Vash1 regulates secondary sprouting

**Fig. 3.**
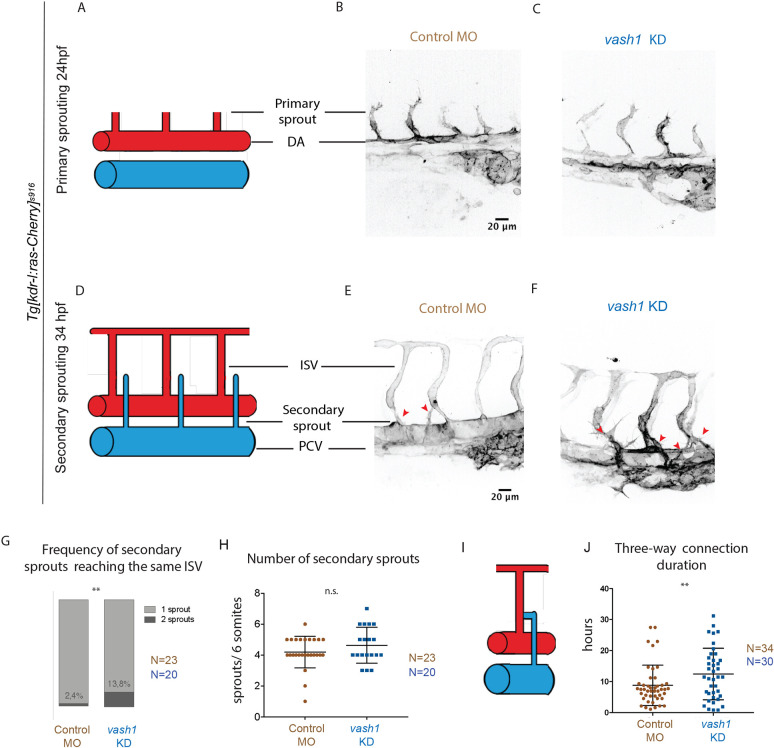
**Vash1 deficient embryos exhibit aberrant secondary sprouting.** (A-F) Primary and secondary sprouting in control and *vash1* KD *Tg[kdr-l:ras-Cherry]^s916^* zebrafish embryos, with labelled blood vessels. Primary sprouting occurs from the DA at 24-30 hpf (A-C). Secondary sprouting occurs from the PCV at 34-40 hpf and ISVs form (D-F). Red arrowheads indicate secondary sprouts (E,F). Pictures are representative of three replicated experiments. (G,H) Quantification of observed frequency that either one or two different sprouts reach an ISV (G) and of number of secondary sprouts per six somites (H). *n*=87 ISVs from 20 embryos for *vash1* KD group, and *n*=82 ISVs from 23 control embryos, from three biological replicates. **P<0.0021 (Mann–Whitney test). n.s., non-significant. (I,J) A transient three-way connection is formed when the secondary sprout connects with the primary ISV and lumenizes (I), after which one of its branches is resolved. The duration of three-way connections from lumenization to resolution was quantified in control and *vash1* MO-injected embryos (J). Data points represent 45 ISVs from 34 embryos for *vash1* KD and 39 ISVs from 30 embryos for control groups, from four biological replicates. **P<0.0021 (Kolmogorov–Smirnov test). Data are median±s.d. (G) and mean±s.d. (H,J).

Live-imaging of sprouting angiogenesis in *Tg[kdrl-l:ras-Cherry;fli1a:nEGFP]* embryos showed comparable primary sprouting and ISV network formation in control and *vash1* KD embryos (Fig. 3A-F). However, secondary sprouting progressed abnormally in *vash1* KD embryos. In control conditions, each secondary sprout finds its ISV, with very few occurrences of two secondary sprouts reaching to the same ISV. Secondary sprouts of *vash1* KD embryos exhibit persistent protrusions, reaching two different ISVs in 13.8% of the cases, in comparison with 2.4% in the controls (Fig. 3G). In search of the underlying mechanism, we asked whether there were more secondary sprouts emerging from the PCV. Quantification revealed a slightly higher number of secondary sprouts in *vash1* KD embryos, but without statistical significance (Fig. 3H). Furthermore, the transient three-way connections (schematic in Fig. 3I) that regularly form during vein/lymphatic development (Geudens et al., 2019) took longer on average to resolve (12.4±1.3 h in *vash1* KD in comparison with 8.9±1 h in the control). In addition, in *vash1* KD embryos, 46% of the three-way connections take longer to resolve than the average, in comparison with 26% of the controls (Fig. 3J), showing a higher variance in the morphants. We hypothesize that there is a disturbed cell migration mechanism, or a surplus of cells in these structures that do not allow for it to resolve as fast. Together, these results suggest that *vash1* regulates secondary sprouting and three-way connection resolution.

### Vash1 regulates cell proliferation

**Fig. 4.**
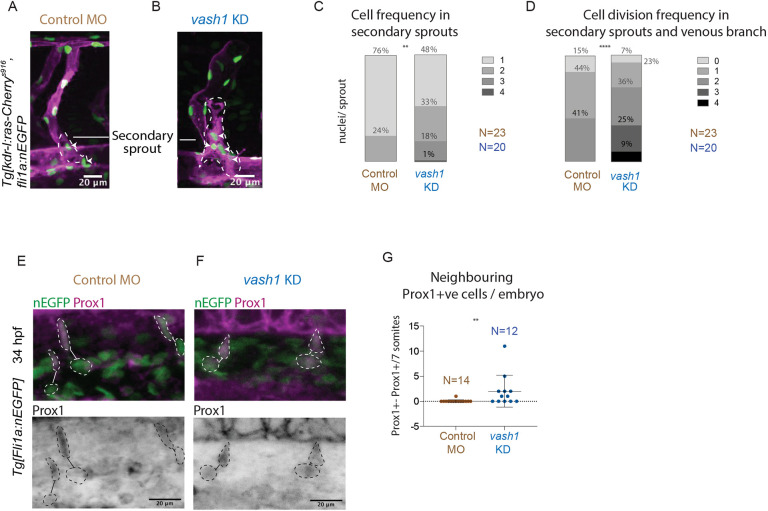
**Secondary sprouts in *vash1* morphants exhibit more Prox1+ cells and higher proliferation rates.** (A,B) Secondary sprouts in *Tg[kdr-l:ras-Cherry^s916^,fli1a:nEGFP^y7^]* embryos, with membrane- and nuclei-labelled endothelial cells (EC) in control (A) and *vash1* KD embryos (B). White arrowheads indicate nuclei in secondary sprouts (outlined by dashed line). (C,D) Quantification of the number of endothelial nEGFP-labelled nuclei in secondary sprouts immediately before connection to the ISV (C), and cell division frequency in the secondary sprout before and after connecting to the ISV, from 30 to 70 hpf (D). Quantifications are from three biological replicates. **P<0.0021, ****P<0.0002 (Mann–Whitney test in C, *t*-test in D). (E,F) Prox1-positive (Prox1+ve) EC were identified by immunostaining in *Tg[fli1a:nEGFPy7]* embryos in control and *vash1* morphants. In controls, migrating cells in secondary sprouts are Prox1+ve and their neighbouring EC in the PCV are Prox1-negative (E). In *vash1* morphants, both EC in the secondary sprout and the neighbouring EC still in the PCV are Prox1+ve (F). Analysed EC are highlighted (dashed line), and neighbouring EC are connected with a line. (G) Quantification of incidence of nEGFP+ Prox1+ve neighbouring EC per seven somites per embryo in both control and *vash1* MO-injected embryos. **P<0.0021 (Mann–Whitney test). Pictures are representative of three replicated experiments.

As the temporary three-way connection resolves by polarized EC migration (Geudens et al., 2019), we hypothesized that disturbed cell migration or a surplus of cells in these structures are causative for its prolonged persistence. Quantification revealed that the *vash1* KD secondary sprouts of *Tg[kdrl-l:ras-Cherry, fli1a:nEGFP]* embryos exhibited an increased number of EC (Fig. 4A,C) compared with control secondary sprouts (Fig. 4B,C). The vast majority (76%) of secondary sprouts of control embryos exhibit one nucleus per sprout, the remaining (24%) have a maximum of two nuclei per sprout. In contrast, only 48% of *vash1* KD sprouts exhibited one nucleus per sprout. The majority (52%) of *vash1* KD sprouts had two to four nuclei. To uncover whether these defects are due to increased cell division we have quantified cell divisions from movies acquired between 30 and 70 hpf at 15 min time resolution and observed twice as many nuclear divisions in the *vash1* KD secondary sprouts than in controls (Fig. 4D; Movies 1 and 2). This difference in proliferation was not detected in EC of the ISVs (Fig. S2A), suggesting that Vash1 negatively regulates cell division during secondary sprouting.

The lymphatic progenitor cells are derived by bi-potential precursors that divide during secondary sprouting into two daughter cells with different prospective identities: venous and lymphatic. As we observe increased secondary sprout cell divisions, we hypothesized that this could impact on the specification of lymphatic EC. To assess the role of Vash1 in this process we performed Prox1 immunostaining in *vash1* morphants at 36 hpf, during sprouting. We observed higher frequencies of Prox1-positive cells in the PCV in *vash1* KD when compared with control MO injected siblings. This suggests that either Prox1 is ectopically induced in PCV or Prox1 expression is retained in both daughter cells after the bi-potential precursor cell division. Quantification of the Prox1-positive nearest neighbour revealed that the Prox1-positive cells in the PCV are always within close proximity of the venous sprout in *vash1* KD embryos, but not in the control MO injected siblings (Fig. 4E-G). These results suggest that Vash1 plays a role in lymphatic progenitor cell specification, potentially by controlling Prox1 distribution in daughter cells post cell division.

### Endothelial microtubules are selectively detyrosinated in secondary sprouts

**Fig. 5.**
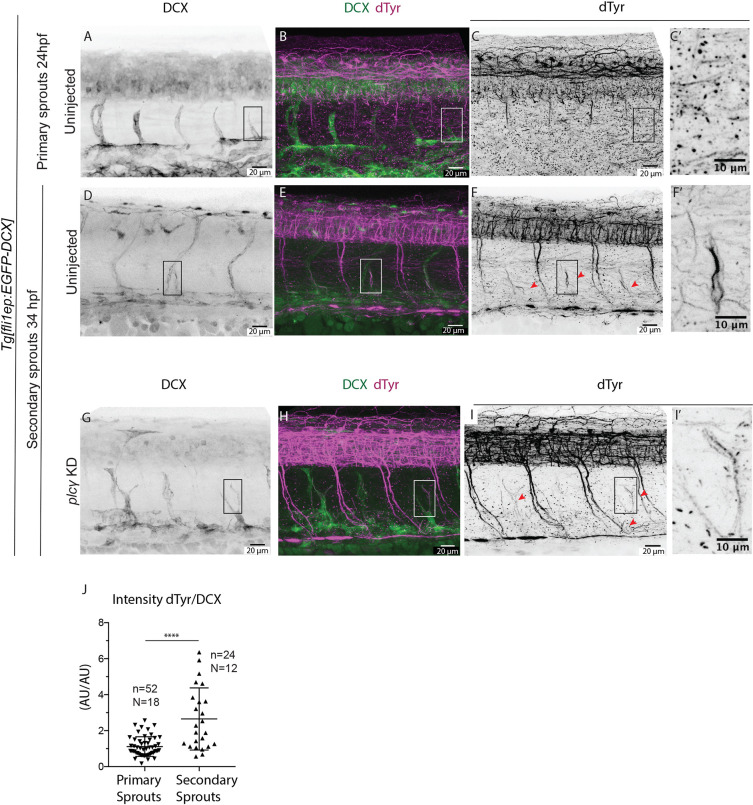
**Microtubules of secondary sprouts are selectively detyrosinated.** (A-I′) Immunostainings using antibody detecting the glutamate amino acid of detyrosinated microtubules (dTyr) during primary (A-C) and secondary (D-I) sprouting in uninjected (A-F) and *plcγ* KD (G-I) *Tg[fli1ep:EGFP-DCX]* embryos, labelling all endothelial microtubules (DCX). C′,F′ and I′ are magnifications from boxed region in C,F,I, respectively. *plcγ* KD embryos (G-I′) show reduced primary sprouting, facilitating the visualization and quantification of dTyr signal specifically in secondary sprouts. Arrowheads indicate secondary sprouts. (J) Quantification of the dTyr/DCX signal intensities in primary sprouts of control MO-injected embryos, and secondary sprouts from *plcγ* MO-injected embryos. *n*=52 control primary sprouts from 18 embryos, *n*=24 *plcγ* KD secondary sprouts from 12 embryos, from one experiment. ****<0.00001 (Mann–Whitney test). Pictures are representative of 3 replicated experiments.

Immunostaining against dTyr in fixed uninjected *Tg[fli1ep:EGFP-DCX]* embryos at 24 and 34 hpf (Fig. 5A-F) revealed that, although the microtubules of the primary sprouts showed little to no staining (Fig. 5B,C,C′), the microtubules of the vast majority of secondary sprout cells were strongly labelled (Fig. 5E,F,F′). Quantification of fluorescence signal in endothelial microtubules from secondary sprouts was challenging owing to the overlap of EC from the primary sprouts. To mitigate this, we used zebrafish *plcγ* (*plcg1*) morphants, which exhibit no primary sprouting and subsequent arterial ISV formation as the VEGFR2-Plcγ-signalling pathway is selectively required for primary spouting (Hogan et al., 2009a). Secondary sprouting, however, proceeds in these embryos, and the absence of ISVs facilitates the measurement of signal intensities in secondary sprouts (Fig. 5G-I). Quantification of the signal intensity ratio between dTyr and DCX confirmed a threefold higher signal in secondary sprouts of *plcγ* morphants than in primary sprouts of control morphants (Fig. 5J). In summary, detyrosinated microtubules appear particularly abundant in secondary sprouts, in comparison with primary sprouts or established ISVs.

### Vash1 regulates formation of trunk lymphatic vasculature

**Fig. 6.**
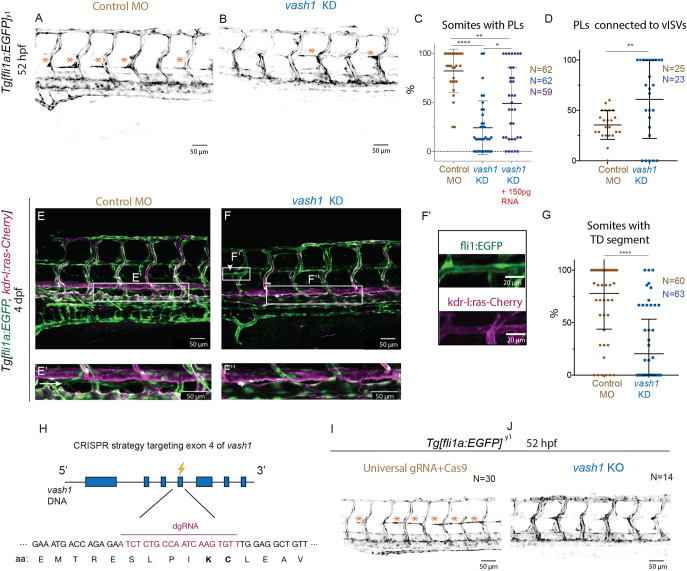
**Vash1 regulates formation of trunk lymphatic vasculature.** (A,B) *Tg[fli1a:EGFP]^y1^* labels parachordal lymphangioblasts (PL, indicated by asterisks) of control (A) and *vash1* KD embryos (B) at 52 hpf. (C) Quantification of the percentage of somites with PLs in embryos injected with control and *vash1* MO, as well as the rescue with 150 pg *vash1* mRNA. Each point corresponds to one embryo. (D) Quantification of percentage of PLs connected to a venous ISV in embryos injected with control and *vash1* MO; 6-8 somites. In C, *n*=62 for controls, *n*=62 for vash1 morphants and *n*=59 for *vash1* morpholino and RNA-injected embryos. In D, *n*=25 for controls and *n*=23 for *vash1* morphants. Three biological replicates were carried out. *P<0.0332, **P<0.02, ****P<0.0001 (Kruskal–Wallis in C, Mann–Whitney test in D). (E-G) Zebrafish trunk of 4 dpf *Tg[kdr-l:ras-Cherry^s916^,fli1a:EGFP^y1^]* zebrafish embryos. Arrowheads indicate GFP- and mCherry-positive putative ISV-to-ISV connections in *vash1* KD embryos (F, ISV-to-ISV connection in F′). The main axial lymphatic in the zebrafish trunk – the thoracic duct (TD) – is GFP-positive, mCherry-negative (E,E′, arrow), and absent in the *vash1* KD embryo (F, ISV-to-ISV connection in F″). The percentage of somites with TD was quantified (G). *n*=60 control and *n*=63 morphants analysed. ****P<0.0001 (Mann–Whitney test). (H) Strategy for CRISPR mutation of exon 4 of *vash1* includes design of a duplex guide RNA (dgRNA) to target the codons that translate into lysine and cystein (in bold) of positions 174 and 175 of the zebrafish Vash1 protein, crucial for the carboxypeptidase function. (I,J) Trunk region of Crispr/Cas injected *vash1* knockout (KO) *Tg[fli1a:EGFP]^y1^* embryos (J) in comparison with the control (I). Embryos injected with control gRNA and Cas9 exhibit regular PL coverage (I, asterisks) and no mutation. *vash1* CRISPants – embryos with confirmed CRISPR mutations – lack PLs (J). *n*=30 control embryos, *n*=14 CRISPants.

Closer examination of *Tg[fli1a:EGFP^y1^,kdr-l:ras-Cherry]* embryos further revealed defects in the formation of lymphatic structures. At 52 hpf, the number of PLs emerging as lymphatic progenitors at the horizontal myoseptum was dramatically reduced in *vash1* KD (24%±27) compared with control embryos (83%±22) (Fig. 6A-C). PLs formation was partially rescued by co-injection with *vash1* mRNA (Fig. 6C), indicating that the observed lymphatic defects are indeed caused by loss of Vash1 function. Defective PL formation was further confirmed by two additional Vash1 MOs (Fig. S3A-E).

In addition, PLs in *vash1* morphants frequently appeared connected to the venous ISVs, unlike in sibling controls (Fig. 6D). Occasionally at 52 hpf, and later in development at 4 dpf, we observed *kdrl-mCherry*-positive lumenised connections between ISVs, formed at the horizontal myoseptum (Fig. 6B,F,F′, arrowhead), suggesting they are aberrant vascular shunts. To assess perfusion of the aberrant ISV-to-ISV connections, we injected *Tg[fli1a:EGFP]^y1^* embryos with fluorescent beads into the general circulation (Qtracker 705 quantum dots) at 4 dpf (Fig. S4B-D) and found that two-thirds of the connections (*n*=10/15) are lumenized, specifically the ones at the horizontal myoseptum.

PLs are transient structures that normally form by sprouting of a subset of the secondary sprouts (Hogan et al., 2009a; Koltowska et al., 2015; Yaniv et al., 2006). From their initial position at the horizontal myoseptum, they migrate ventrally to shape the main trunk lymphatic vessel, the TD (Bussmann et al., 2010). The TD can be detected in *Tg[fli1a:EGFP,kdr-l:ras-Cherry]* embryos as an eGFP-positive and mCherry-negative vessel that locates between the DA and the PCV at 4 dpf (Fig. 6E). Control embryos showed almost complete TD formation along the trunk, whereas in *vash1* morphants the TD was mostly absent (Fig. 6E′,F″). Quantification revealed a significant reduction in the percentage of somites with a TD fragment in *vash1* KD embryos (20%±4.18) compared with controls (78%±4.4) (Fig. 6G). In addition, at this developmental stage, we observed a variable degree of under-development of the intestinal vascular system in the *vash1* morphants (Fig. S6A-I).

For further validation of the *vash1* morphant phenotypes, we generated F0 CRISPR mutants (CRISPants) with a dgRNA targeting exon 4 of *vash1* at which two codons translate into lysine (K) and cysteine (C) at positions 174 and 175 of Vash1 (Fig. 6H). These amino acids are crucial for the carboxypeptidase function of Vash1 (Adamopoulos et al., 2019; Nieuwenhuis et al., 2017) (Fig. 1B). Imaging 60 injected embryos revealed decreased PL formation in the trunk of 14 embryos (Fig. 6I). Sequencing of the targeted exon-4 of the *vash1* locus in these 14 embryos revealed mutations (specified in Fig. S5A) affecting either both lysine 174 and cysteine 175, or only cysteine 175 in all CRISPants, but not control-injected embryos. Given the essential function of these residues, the results suggest that the mutant Vash1 in CRISPants cannot detyrosinate microtubules. In contrast, embryos injected with control gRNA and Cas9 exhibited regular PL coverage and no mutations. Together, these results indicate that the reduction of the PL coverage is a result of a lack of tubulin detyrosination as a consequence of either *vash1* KD in morphants or mutation in *vash1* CRISPants, respectively. In summary, *vash1* zebrafish morphants and mutants show a decrease in lymphatic progenitors and, as a consequence, a decrease in mature lymphatic vasculature of the trunk.

## DISCUSSION

Tubulin detyrosination is important for several differentiation events and developmental processes, such as myogenesis (Chang et al., 2002; Gundersen et al., 1989; Kerr et al., 2015) and neurogenesis (Aillaud et al., 2017; Erck et al., 2005), although the cellular mechanisms behind this remain poorly understood. Vash1 was recently identified as the catalyst of this reaction (Aillaud et al., 2017; Nieuwenhuis et al., 2017). Vash1 is also anti-angiogenic. The cellular mechanisms behind the vascular phenotypes that are caused by Vash1 loss- and gain-of-function are not well understood.

**Fig. 7.**
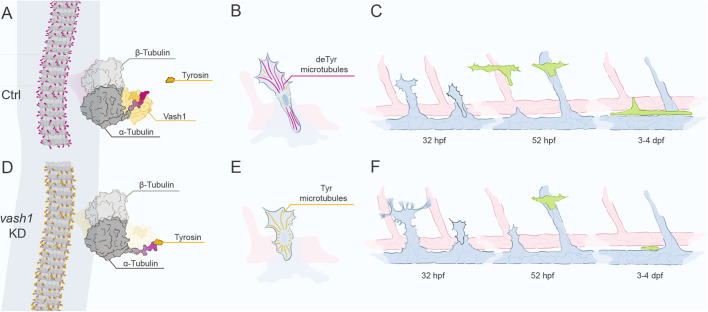
**Model of Vash1 function in zebrafish trunk.** (A-F) Microtubule detyrosination is catalysed by Vash1 (A), particularly occurring in secondary sprouts (B). In the secondary sprouts, Vash1-mediated detyrosinated microtubules keep control of the number of sprouting cells, cell proliferation and cell protrusion formation to avoid over-sprouting (C). Secondary sprouts with correctly detyrosinated microtubules form PLs at 2 dpf, which in turn form the TD at 3-4 dpf (C). Upon *vash1* KD, microtubules of secondary sprouts are no longer detyrosinated (D,E). Cells of secondary sprouts over-proliferate, fail to form PLs and a functional TD, although PLs forming from veins still occur (F).

In this study, we identified Vash1 as a novel regulator of EC sprouting from the PCV and the subsequent formation of lymphatic vessels in the zebrafish trunk. We showed that microtubules in secondary sprouts are particular in that they are highly detyrosinated by Vash1 (Fig. 7A-C). We further show that *Vash1* is dynamically expressed in the zebrafish in the endothelium of the DA and in perivascular tissues during both sprouting waves. This is in accordance with earlier reports of *Vash1* being highly expressed in angiogenic vessels, healthy or tumour associated, *in vitro* and *in vivo* (Watanabe et al., 2004) as well as in arteries (Shibuya et al., 2006). Previous research also showed that, despite its induction by VEGF, Vash1 is mostly expressed in the quiescent endothelium, adjacent to the sprouting front (Kimura et al., 2009), potentially to control the amount of sprouting cells.

Our present study in the zebrafish demonstrated that depletion of Vash1 reduces the level of microtubule detyrosination and negatively affects secondary sprouting (Fig. 7D,E). Sprouts emerging from the PCV in *vash1* morphants contain more endothelial nuclei, as EC divide almost twice as often as in control conditions. Furthermore, aberrant protrusions from these sprouts reach neighbouring ISVs almost six times more often than in controls. These results suggest that Vash1-driven microtubule detyrosination limits excessive venous EC sprouting behaviour and proliferation during lymphovenous development in zebrafish (Fig. 7F). However, given that Vash1 may also have additional functions beyond its carboxypeptidase activity driving tubulin detyrosination, the KD results alone do not demonstrate causality. The fact that we observe a similar defect in PL formation in F0 CRISPants carrying mutations that disrupt its catalytic function, however, strongly suggests that microtubule detyrosination is indeed the mechanism required for adequate lymphovenous patterning. Nevertheless, future work will need to clarify exactly how microtubule detyrosination controls secondary sprouting and lymphatic specification.

Although the cellular mechanisms of primary and secondary sprouting in zebrafish appear to be very similar (Isogai et al., 2003), secondary sprouting uses alternative signalling pathways and entails a unique specification step that establishes both venous ISVs and lymphatic structures. Secondary sprouting in zebrafish is triggered by Vegfc and activation of its receptor Flt4 in a subpopulation of EC in the PCV (Bussmann et al., 2010; Hogan et al., 2009b) that increase *prox1a* expression before a cell division (Koltowska et al., 2015; Nicenboim et al., 2015). This cell division is thought to lead to distinct identity acquisition by the daughter cells, as one Prox1-negative cell remains in the PCV while the other Prox1-positive cell sprouts from the PCV to ultimately become a lymphatic progenitor (Koltowska et al., 2015; Nicenboim et al., 2015). The specification of lymphatic progenitors is influenced by the levels of *prox1a* expression, with high *prox1a* expression thought to favour lymphatic specification (Koltowska et al., 2015). In *vash1* morphants, the observed increase of neighbouring cells with *prox1a* expression suggests a retention of the Prox1 protein in both daughter cells upon cell division. This highly aberrant phenotype is reminiscent of what has been published for embryos with *vegfc* overexpression (Koltowska et al., 2015), suggesting that the differential activity of the Vegfc-Flt4 signalling axis is possibly perturbed upon *vash1* KD. In the case of *vegfc* overexpression, this is consistent with a disruption of *prox1a* in the daughter cells, increase of cell divisions and disruption of the asymmetric cell behaviour of the daughter cells.

Intriguingly, microtubule detyrosination has been shown to influence mitotic and meiotic spindle (Akera et al., 2017; Barisic et al., 2015; Liao et al., 2019), with emerging evidence for a potential role in asymmetric cell fate (Akera et al., 2017; Laloraya, 2018). Given recent discoveries of asymmetric cell fate acquisitions and behaviour upon cell division in EC (Costa et al., 2016; Koltowska et al., 2015; Nicenboim et al., 2015), it is tempting to speculate that the lack of detyrosinated microtubules interferes with this process during initiation and progression of secondary sprouting, such that endothelial daughter cells retain their undifferentiated state and fail to specialise into lymphatic EC. Failure to differentiate under activated conditions is frequently associated with continued proliferation, also in EC (Benedito et al., 2008; Siekmann and Lawson, 2007). A recent study reported a similar increase in secondary sprout numbers in the *nova2* zebrafish mutants (Baek et al., 2019). Nova2 acts as a splicing factor that limits Vegfc/Flt4 signalling. Its absence causes premature and excessive formation of sprouting venous EC that fail to fully differentiate into functional lymphatic structures owing to an increase of Vegfc/Flt4/Mapk signalling and consequently persistent proliferation.

According to current understanding, the decisive fate-determining step in the formation of the lymphatic system in the zebrafish trunk is the acquisition of a *prox1a* high expression status in a few cells of the PCV. These cells emerge from the PCV as secondary sprouts to form first the PLs and subsequently expand to form the TD and other trunk lymphatic vessels (Koltowska et al., 2015; Nicenboim et al., 2015). Recent work demonstrated that a secondary sprout either contributes to remodelling a pre-existing ISV into a vein, or forms a PL. In addition, a single secondary sprout is able to simultaneously form a venous ISV and give rise to lymphatic progenitors, which can be seen emerging from a remodelled venous ISV (Geudens et al., 2019; Isogai et al., 2003). Unlike in control siblings, the few PLs forming in *vash1* morphants are found associated with venous ISVs and often have a more vascular appearance, suggesting a defect in lymphatic specification. In addition, the number of PLs present appears to be insufficient to form the trunk lymphatic vasculature. The expressivity of this morphant phenotype is variable, suggesting that the formation of PLs and lymphatics may be dynamically regulated and affected by variations in KD efficiency and thus Vash1 levels. Interestingly, homozygous and heterozygous Vash1 mouse mutants show differences in their vascular phenotype upon injury (Kimura et al., 2009), suggesting a dose-dependent effect of Vash1.

Vash1 is highly conserved across eukaryotes, especially the three amino acids that constitute a non-canonical Cys-His-Ser catalytic triad active site (Sanchez-Pulido and Ponting, 2016) that are necessary for the tubulin detyrosination function (Adamopoulos et al., 2019; Nieuwenhuis et al., 2017). Human and murine VASH1 protein variants induce similar effects in angiogenesis assays when applied ectopically (Watanabe et al., 2004), suggesting similar functions across these species. Our pairwise sequence analysis indicates that the residues necessary for tubulin recognition and detyrosination (Adamopoulos et al., 2019; Nieuwenhuis et al., 2017) are conserved between human and zebrafish versions of Vash1, suggesting conserved carboxypeptidase function. The fact that we could observe absence of microtubule detyrosination in zebrafish upon *vash1* KD further supports this hypothesis.

Although earlier studies using gain-of-function approaches reported anti-lymphangiogenic activities of ectopically added VASH1 in the mouse cornea and in tumour conditions (Heishi et al., 2010), our loss-of-function analysis in zebrafish suggests a selective pro-lymphangiogenic function of Vash1 by supporting lymphovenous specification. Although this appears to be contradictory at first sight, a possible influence on Vegfc-mediated effects may explain the discrepancy. The ectopic VASH1 might act as an acute inhibitor of Vegfc signalling in existing lymphatic structures such as cornea or tumour, thus inhibiting further lymphatic growth, whereas the absence of Vash1 during secondary sprouting resembles excessive Vegfc signalling and interferes with lymphatic differentiation. Future work will need to establish whether and how Vash1-mediated detyrosination of endothelial microtubules restricts excessive Vegfc signalling, in some or all daughter cells, during formation of secondary sprouts from the PCV.

In conclusion, our work identifies Vash1 as a conserved tubulin carboxypeptidase in zebrafish, with a selective role in secondary sprouting and lymphatic cell formation. We propose that a balance of the number of EC sprouting from the cardinal vein is maintained by Vash1 via microtubule detyrosination and consequent EC divisions and that Vash1 has a role in lymphatic specification and formation in the zebrafish trunk.

## MATERIALS AND METHODS

### Zebrafish maintenance and transgenic lines

Zebrafish (*Danio rerio*) were raised and staged as previously described (Kimmel et al., 1995). For growing and breeding of transgenic lines, we complied with regulations of the animal ethics committees at the Max Delbrück Center for Molecular Medicine, Berlin (Aleström et al., 2019). The following transgenic lines were used: *Tg[fli1a:EGFP]^y1^* (Lawson and Weinstein, 2002) labels all EC, *Tg[fli1a:nEGFP]^y7^* labels all EC nuclei (Roman et al., 2002), *Tg[kdr-l:ras-Cherry]^s916^* (Hogan et al., 2009a) labels EC membrane and *Tg[fli1ep:EGFP-DCX]* (Phng et al., 2015) labels endothelial doublecortin, a microtubule-associated protein.

### dTyr immunofluorescence staining and imaging

Embryos were treated with 1-phenyl-2-thiourea (PTU) at stages later than 24 hpf to prevent pigment formation. Embryos were fixed with 4% paraformaldehyde (PFA) for 10 h at 4°C, and kept for a maximum of 4 days in phosphate-buffered saline (PBS) with 0.5% Triton X-100 (PBTX) at 4°C. Embryos were permeabilized by dehydrating them in a methanol series – 25%, 50% and 75% –and incubated for 1 h at −20°C, before being rehydrated in a descending methanol series – 75%, 50% and 25% – and washed three times with PBTX and post-fixed with 4% PFA for 10 min. The embryos were incubated for 2 h in blocking buffer (10% horse heat inactivated serum, 1% DMSO, 100 mM maleic acid in 0.1% PBTX) at room temperature. Embryos were incubated at 4°C overnight with primary antibodies (indicated below) diluted in blocking buffer. The embryos were then washed with a solution containing 1% DMSO and 100 mM maleic acid in 0.1% PBTX at room temperature and blocked again for 30 min before being incubated with secondary antibodies diluted in blocking buffer overnight. Embryos were washed again in 1% DMSO and 100 mM maleic acid in 0.1% PBTX, fixed with PFA 4% for 30 min at room temperature, washed again and stored at 4°C in PBST until being mounted in glycerol and stored at 4°C.

Primary antibodies used were anti-detyrosinated tubulin (1:600, rabbit; Liao et al., 2019) and anti-GFP FITC conjugate (1:200, mouse, Abcam, ab6662). The secondary antibody used was Alexa 568 against rabbit (1:200, donkey, Invitrogen, A10037).

### dTyr immunofluorescence quantifications

Substacks containing single ISVs were cropped manually from images of whole *Tg[fli1ep:EGFP-DCX]* embryos stained with dTyr antibody. To estimate the background, which varied locally owing to autofluorescence of the embryos, each image was filtered with a large Gaussian filter (radius=50 pixels) and subtracted from the original image. A maximum intensity projection of the DCX channel was used to manually segment endothelial microtubules, with particular care taken to exclude the neurons. The resulting mask was applied to background corrected, sum intensity projections of DCX and dTyr channels. Mean intensity was extracted and plotted to quantify the normalised and direct differences of tubulin detyrosination intensity in different experimental groups. The analysis was performed in Fiji. For all quantifications a minimum of three biological replicates was carried out per quantification.

### Prox1 immunofluorescence staining, imaging and quantification

Whole-mount Prox1 immunofluorescence was performed as previously described (Koltowska et al., 2015; Shin et al., 2017) in *Tg[fli1a:nEGFP]^y7^*. Antibodies used were chicken α-GFP (1:400, Abcam, ab13970) rabbit α-Prox1 (1:500, AngioBio Co, 11-002P), α-rabbit IgG-HRP (1:1000, Cell Signaling Technology, BioNordika, 7076S) and TSA-Cy3 (1:50, PerkinElmer, Akoya, NEL744001KT). The trunks of zebrafish were cut at the junction of the yolk and the yolk extension and flat mounted in clearing solution Omnipaque™ (350 mg I/ml iohexol, GE Healthcare) on a glass-bottom imaging dish. The images were taken with the inverted confocal Leica SP8 DSL system using a 25× water objective.

Prox1 and nEGFP double-positive cells were identified in the PCV and secondary sprouts. Neighbouring cells were defined by adjacent nEGFP+ EC, normally corresponding to a cell in the PCV and the other in the secondary sprout. When two neighbouring EC expressed Prox1, the occurrence was quantified. The number of incidences was counted per seven somites in every embryo, in three biological replicates.

### *In situ* hybridization

Primers used to amplify templates for riboprobe production were designed to span the exon/exon junction: GAGACCTGCCCAAGATTCCAG and GGCCGCTTCTCTACTGATGG. The anti-sense transcript was transcribed *in vitro* using 20 U/μl T7 polymerase (Promega). The transcription was carried out with 5× transcription buffer (Promega, P118B), DTT 0.1 M (Promega, P1171), digoxigenin labelling mixes (Roche) and 40 U/μl RNAsein (Promega) for 2 h at 37°C. The plasmid was removed by adding 2 μl of RNase-free DNase I and 18 μl sterile water, mixed and incubated for 30 min at 37°C. The RNA product, the riboprobe, was purified using the RNA easy kit (Qiagen) and diluted to 1 ng/μl in Hyb+ buffer (Thisse and Thisse, 2008).

The *in situ* hybridizations were performed as previously described (Thisse and Thisse, 2008; Habeck et al., 2002), with treatments at the following concentrations of proteinase K in PBST (PBS, 0.1% Tween-20): at 24 hpf, 0.01 mg/ml, incubated for 5 min; at 34 hpf, 0.01 mg/ml, incubated for 8 min; at 48 hpf, 0.02 mg/μl, incubated for 30 min. The embryos were incubated in NBT/BCIP staining solution (Roche) overnight at 4°C. The reaction was stopped by washing with PBST three times, fixed for 20 min in PFA, dehydrated in methanol, and mounted and imaged within a week, in glycerol. The imaging was performed using a Zeiss Stemi CV11 stereoscope equipped with an AxioCam and processed using the software AxioVision Rel. 4.5.

### Live imaging and analysis

Embryos were dechorionated and anaesthetised with 0.014% (1×) tricaine methanesulfonate (MS-222, Sigma-Aldrich). Embryos were then mounted in 0.8% low melting point agarose (Life Technologies) containing 0.014% tricaine and immersed in E3 buffer containing 0.014% tricaine and 0.003% PTU when indicated. Live embryos were imaged using an upright 3i Spinning Disk Confocal Microscope using Zeiss Plan-Apochromat 20× and 40×/1.0 NA water dipping objectives. Image processing was performed using Fiji software (Schindelin et al., 2012). XY drifts were corrected using the MultiStackReg plugin (from B. Busse, Eunice Kennedy Shriver National Institute of Child Health and Human Development, USA).

### Secondary sprout and three-way connection parameters

The number of secondary sprouts was quantified in 15 min time-lapse movies from 24 to 48 hpf, in *Tg[kdrl-l:ras-Cherry,fli1a:nEGFP]* embryos. The number of nuclei was assessed in each secondary sprout immediately before connection to the pre-existing ISV. Cell divisions in secondary sprouts were quantified by adding nuclear (nEGFP) divisions from the time of emergence, until the resolution of the three-way connection with pre-existing ISVs. The duration of the three-way connection was quantified in the same embryos from the moment it connected to the primary ISV and lumenised until the three-way connection was resolved. For all quantifications a minimum of three biological replicates was carried out per quantification.

### Lymphatics: PL and TD quantifications

PL and TD were quantified by examining *z*-stacks of 52 hpf and 4 days post fertilization (dpf) *Tg[fli1a:EGFP^y1^]* embryos and counting the presence or absence in each somite, starting from the fifth-most anterior ISV. The number of PL associated with venous ISVs were identified by a connection to a venous ISV at 52 hpf in *vash1* morphants and control siblings. For all quantifications a minimum of three biological replicates was carried out per quantification, using six to eight somites per embryo.

### Statistical analysis

Zebrafish embryos were selected on the following pre-established criteria: normal morphology, beating heart and flowing blood. All morphant embryos analysed in this project exhibited no apparent visual phenotype. The experiments were not randomised and the investigators were not blinded during experiment and analysis.

In order to decide whether a parametric or non-parametric test should be used to compare different experimental groups, we assessed whether the population was normally distributed using an Anderson-Darling test. If so, *t*-test and regular ANOVA were performed, if not, Mann–Whitney and Kruskall–Wallis tests were performed. Data are presented in all graphs as mean±s.d., except in Fig. 3G in which they are median±s.d.

### Cell isolation for gene expression analysis

*Tg[fli1a:nEGFP]^y7^* were outcrossed and their embryos collected. Negative embryos were also collected under the stereoscope, to serve as GFP-negative control siblings during cell sorting. Embryos were treated with 0.003% PTU when older than 24 hpf. The embryos were dechorionated by adding pronase (1 mg/ml) for 15 min, washed in E3 buffer and anaesthetised using 0.16 mg/ml tricaine methanesulfonate (Sigma-Aldrich). Then 250 embryos were transferred to a 1.5 ml tube and the yolk sac was removed by two rounds of centrifugation (or until the solution was clear) with calcium ringer solution at 2000 rpm (400 ***g***) at 4°C for 5 min. The embryos were then resuspended in 1 ml of protease solution [0.07 mg/ml of liberase in Dulbecco's PBS (DPBS); Thermo Fisher Scientific] with 0.4 U/ml DNAse I (Invitrogen). The embryos were dissociated at 28°C for 10 min on a rotator (or until the embryos appeared visually digested to a single cell solution), pipetting up and down with a 200 ml tip every 4-5 min. The reaction was stopped by placing the cell solution on ice and adding CaCl_2_ to a final concentration of 1-2 mM with 0.5% fetal bovine serum (FBS) in DPBS. The cells were centrifuged at 2000 rpm for 5 min at 4°C, the supernatant was discarded, and the pellet resuspended in 1 ml resuspension solution (DPBS with 2 mM EDTA, 0.4 U/ml DNAseI and 0.5% FBS) to avoid cells clumping together. The cells were passed through a 40 μM strainer into to a 50 ml falcon tube, following a pre-wash of the strainer with 500 μl of the resuspension solution. Another 500 μl of the resuspension solution was added to the filter to remove any remaining cells. The cells were centrifuged at 2000 rpm for 5 min at 4°C and resuspended in 500 μl of DPBS+EDTA solution. The cell solution was transferred to a 5 ml polypropylene (Falcon) dedicated test tube and kept on ice until sorting with a FACSAria™ III Sorter (BD). Cells were sorted and filtered against triplets and droplets. Negative control cells from negative nEGFP siblings were used to define the GFP+ collection window, to filter against auto-fluorescence. nEGFP-positive and -negative cells from green *Tg[fli1a:nEGFP]^y7^* embryos were collected in different 1.5 ml Eppendorf tubes with 350 μl Trizol.

### RNA extraction

The cell lysis suspension was vortexed for 15 s, spun down and incubated at room temperature for 5 min and 0.5 μl glycoblue, a nucleic acid coprecipitant, was added to the lysed solution to aid visualization of the precipitated RNA. Then 50 μl chloroform was added to each tube, the tubes were shaken vigorously for 15 s and incubated at room temperature for 5 min. Tubes were centrifuged at 12,000 rpm (14,489 ***g***) for 15 min at 4°C. RNA was present in the transparent supernatant. The supernatant was carefully pipetted to a LoBind Eppendorf tube, with 125 μl added to the aqueous phase and the tube shaken vigorously for 15 s. The solution was incubated at −20°C overnight. The solution was then centrifuged at 12,000 rpm for 10 min at 4°C. The supernatant was then removed, leaving the small blue RNA pellet, which was washed with 500 μl of 75% ethanol, shaken and centrifuged at 7500 rpm (5660 ***g***) for 5 min at 4°C. The supernatant was discarded and the pellet air dried until no liquid was left. The pellet was resuspended in 13 μl of DEPC-treated water and the concentration and purity were measured using a Nanodrop 2000 (Thermo Fisher Scientific), and the reverse transcription was carried out for samples presenting with an RNA concentration above 20 ng/μl and a 260/280 purity higher than 1.8 using the Superscript IV First-Strand cDNA synthesis reaction kit (Thermo Fisher Scientific) and random hexamer primers. The reaction was incubated for 1 h. The final reaction was diluted at 1:10 and kept at −20°C.

### qPCR

The primers were designed using Primer blast (http://www.ncbi.nlm.nih.gov/tools/primer-blast/) to have a melting temperature of 60°C and to span exon-exon junctions to avoid amplification of genomic DNA. Each reaction was set in a 20 μl volume mix containing 10 μl of SYBR-green qPCR mix (Roboklon), 0.8 μl cDNA (1:50 dilution), 0.45 μl of carboxyrhodamine (ROX) and 0.25 μl uracil-N-glycosylase (UNG) from Roboklon and primers to a final concentration of 300 μM. Three technical replicates of each sample were used. Amplification was performed in triplicates in 384-well plates (Applied Biosystems) with the following thermal cycling conditions: initial uracil-DNA glycosylase (UDG) treatment 50°C for 10 min, followed by 40 cycles of 15 s at 95°C and 60 s at 60°C. Control reactions included a no template control (NTC). qPCR was performed using a Quantstudio6Flex machine (Life Technologies). Amplification curves were analysed to confirm the presence of a single PCR product, and outliers excluded when one of the Ct values was very different from the other two. qPCR data was analysed using the Pfaff method (Pfaffl, 2001) that integrates primer amplification efficiency, determined by LinReg software (Ruijter et al., 2009). Samples were normalised against four endothelial housekeeping genes: *rpl13*, *rps29*, *hprt1* and *eef1a1l1*. The geometric average of the housekeeping genes was used for normalization of gene expression (Coxam et al., 2014). Three replicates of the qPCR were carried out. Primers used were: *vash1*-1 fw GGGACATGAGGCTCAAGATTGG, rev GTCGCTCTGGCTGTTCTTGC; *vash1*-2 fw ATCATTAACAGGGGCGGCCT, rev GGTACTGGAATCTTGGGCAGGT; *rpl13* fw CATCTCTGTTGACTCACGTCG, rev CATCTTGAGCTCCTCCTCAGTAC; *rps29* fw TTTGCTCAAACCGTCACGGA, rev ACTCGTTTAATCCAGCTTGAC; *hprt1* fw ATCATGGACCGAACTGAACG, rev AGCGATCACTGTTGCGATTA; *eef1a1l1* fw CTGGAGGCCAGCTCAAACAT, rev ATCAAGAAGAGTAGTACCGCTAGCATTAC.

### Protein extraction for protein analysis

We dechorionated 35 embryos by adding pronase (1 mg/ml) and anaesthetising with 0.16 mg/ml tricaine methanesulfonate (Sigma-Aldrich). Yolk sacs were removed with two rounds of centrifugation in Calcium ringer solution at 2000 rpm (400 ***g***) at 4°C for 5 min each and all liquid was removed until only a dry pellet of embryos was left in the tube. Pellets were fast frozen with liquid nitrogen and kept at −80°C for at least 20 min. The protein extraction procedure included adding 40 μl of lysis buffer (1 M Tris-HCl, 0.5 M EDTA, 10% Brij 96, 10% NP-40) and 0.4 μl of protease inhibitor cocktail (Thermo Fisher Scientific), homogenization with a pestle (Starlab) and centrifugation at 4°C for 15 min. The concentration of the protein extracts was measured using the BCA protein assay kit (Thermo Fisher Scientific).

### Western blot

We mixed 50 μg of the lysates with reducing sample buffer (1:4) and heated the mixture for 5 min at 95°C to denature proteins. The proteins were electrophoresed for 1 h at 150 V and subsequently transferred onto MtOH activated polyvinylidene fluoride membranes (GE Healthcare). Equal loading was assessed using Ponceau Red solution. Membranes were blocked with blocking solution [5% non-fat dry milk in Tris-buffered saline with 0.1% Tween^®^ 20 detergent (TBS-T)] for 1.5 h at room temperature and then incubated with primary antibody against Vash1 (1:500, Proteintech, 12730-1-AP) overnight at 4°C. After incubation with primary antibody, the membranes were washed four times in TBS-T and then incubated with a secondary antibody for 1 h at room temperature (ECL anti-rabbit IgG HRP, 1:4000; GE Healthcare, #NA934V) and washed three times with TBS-T. Immunodetection was performed using a chemiluminescence kit (SuperSignal West Dura; Pierce), and bands were developed using the Las-4000 imaging system (Thermo Fisher Scientific). After initial immunodetection, membranes were stripped of antibodies using the Stripping kit (Thermo Fisher Scientific) at 56°C for 40 min and re-probed with anti-β-actin antibody for 1 h (1:1000, Sigma-Aldrich, A5441). The membranes were washed in TBS-T and incubated for 3 h at room temperature with a secondary antibody (ECL anti-mouse IgG HRP, 1:5000, GE Healthcare, #NA931V). Three replicates of experiments per western blot were performed.

### Morpholino experiments

The morpholino against *plcγ* was used as previously described (Hogan et al., 2009a). Upon injection with morpholinos targeting *Vash1*, a portion of the embryos is morphologically aberrant (Fig. S1E,F). Only embryos with normal looking morphology were taken for further analysis (Fig. S1A-D). Dosage curves were used to find the appropriate dosage for injection (indicated below; Fig. S1E,G,H,I).

The injection mix used 0.4% phenol red, and 1 nl was injected at the one-cell stage. The amount of control MO injected was the same as the total amount of experimental MO injected. *vash1* morpholino 1, 5′-ATTAATCTGAGGAGCACACGGCAGT-3′ (3 ng injected); *vash1* morpholino 2, 5′-GTAACTGAGCCATCGCAGGAGTTAA-3′ (5 ng injected); *vash1* morpholino 3, 5′-CAGGACACCGGCATCAGCAGAACAC-3′ (2 ng injected); *plcγ* morpholino, 5′-ATTAGCATAGGGAACTTACTTTCG-3′ (10 ng injected); control morpholino, 5′-CCTCTTACCTCAGTTACAATTTATA-3′ (same as experimental MOs).

### Rescue experiment

*vash1* full-length cDNA (EMSEMBL ENSDART00000143819.3) was designed and synthesised by GenScript. TA overhangs were added by incubating the insert for 10 min at 72°C with 50 mM DNTPs and Taq polymerase (New England Biolabs), in order to perform TA cloning into a PCR4 TOPO vector (Thermo Fisher Scientific). Integration was confirmed by sequencing. In order to transcribe the cDNA into RNA, the construct was linearized using NotI and the cDNA was transcribed for 4 h by Megascript kit (Life Technologies) using the T3 promoter. The RNA was purified by lithium-chloride precipitation to a concentration of 500 ng/μl. Aliquots were kept at −80°C until further use. For rescue experiments, 1 nl of 150 pg RNA was injected into embryos already injected with MOs against *vash1* or control.

### CRISPR/Cas mutagenesis

The target specific Alt-R CRISPR RNA (crRNA) was designed and ordered at https://eu.idtdna.com/, to target exon 4 of *vash1*, in which two codons translate into lysine (L168) and cysteine (169C), crucial for the carboxypeptidase function of Vash1 (Adamopoulos et al., 2019). The crRNA was annealed with the common Alt-R transactivating crRNA (tracrRNA) from IdtDNA, as previously described (Hoshijima et al., 2019). To synthesize spCas9 protein, a pET-28b-Cas9-His plasmid (Addgene plasmid #47327) was transformed into *Escherichia coli* Rosetta(DE3)pLysS cells (Merck Millipore, 70956-3). Cells were pelleted incrementally at 4000 rpm (3041 ***g***) at 4°C for 10 min. The pellet was resuspended in 30 ml wash buffer. Cells were sonicated for 3× 15 s with a break of 1 min each time at cycle five, amplitude 92%. The resulting cell suspension was centrifuged for 1 h at 14,000 rpm (37,252 ***g***) to pellet the cell debris. The His-tagged Cas9 was extracted using nickel resin columns.

The injection mix was composed of 1 μl 25 μM gRNA (targeting *vash1* or universal control), 1 μl 25 μM Cas9 stock, 1.75 μl H_2_O, 0.75 μl 2 M KCl and 1 μl 0.25% Phenol Red solution. Before microinjection, the solution was incubated at 37°C for 5 min and then placed on ice, and then ∼1 nl was injected. The crRNA targeting exon 4 of *vash1* was ATCTCTGCCAATCAAGTGTTT, and the universal guide RNA (Wierson et al., 2020) was GGGAGGCGTTCGGGCCACAGCGG

### Genotyping

DgRNA- and Cas9-injected embryos were imaged using the confocal spinning disk microscope at 52 hpf for PL phenotyping, as previously described. Afterwards, genomic DNA was extracted from individual embryos at 2 dpf: dechorionated embryos were incubated in 40 μl 50 mM NaOH at 95°C for 10 min. After cooling to 4°C, 13.6 μl of 0.5 M Tris-HCl (pH 8.0) was added to neutralize. A PCR with forward (ATCTCTGCCAATCAAGTGTTT) and reverse (AGCAGCACATTTCACTGCAGA) primers was performed, as well as an incubation of 8 min at 72°C with a Taq polymerase to add AAA overhangs. The PCR products were subsequently confirmed in an agarose gel, gel-purified using a Promega DNA clean-up and concentration kit (#A2893) and TA-cloned into a PCR4 vector. To understand the frequency of mutated cells in a single embryo, five clones per embryo were sequenced.

### Perfusion assay

The Qtracker 705 quantum dots solution (Invitrogen, Q21061MP) was injected in the heart of 4 dpf zebrafish embryos anaesthetized with 0.014% tricaine and mounted laterally in 0.8% low-melting agarose. The quantum dots were excited with a 561 nm laser and their emission detected between 665.5 and 735.5 nm. For all quantifications, a minimum of three biological replicates were used.

## Supplementary Material

Supplementary information

Reviewer comments
