# Supplementary material for: Vasohibin 1 selectively regulates secondary sprouting and lymphangiogenesis in the zebrafish trunk
Source: Development. 2021 Feb 19;148(4):dev194993. doi: 10.1242/dev.194993 (PMC7904002; doi:10.1242/dev.194993)
Supplement: Supplementary information [file develop-148-194993-s1.pdf]

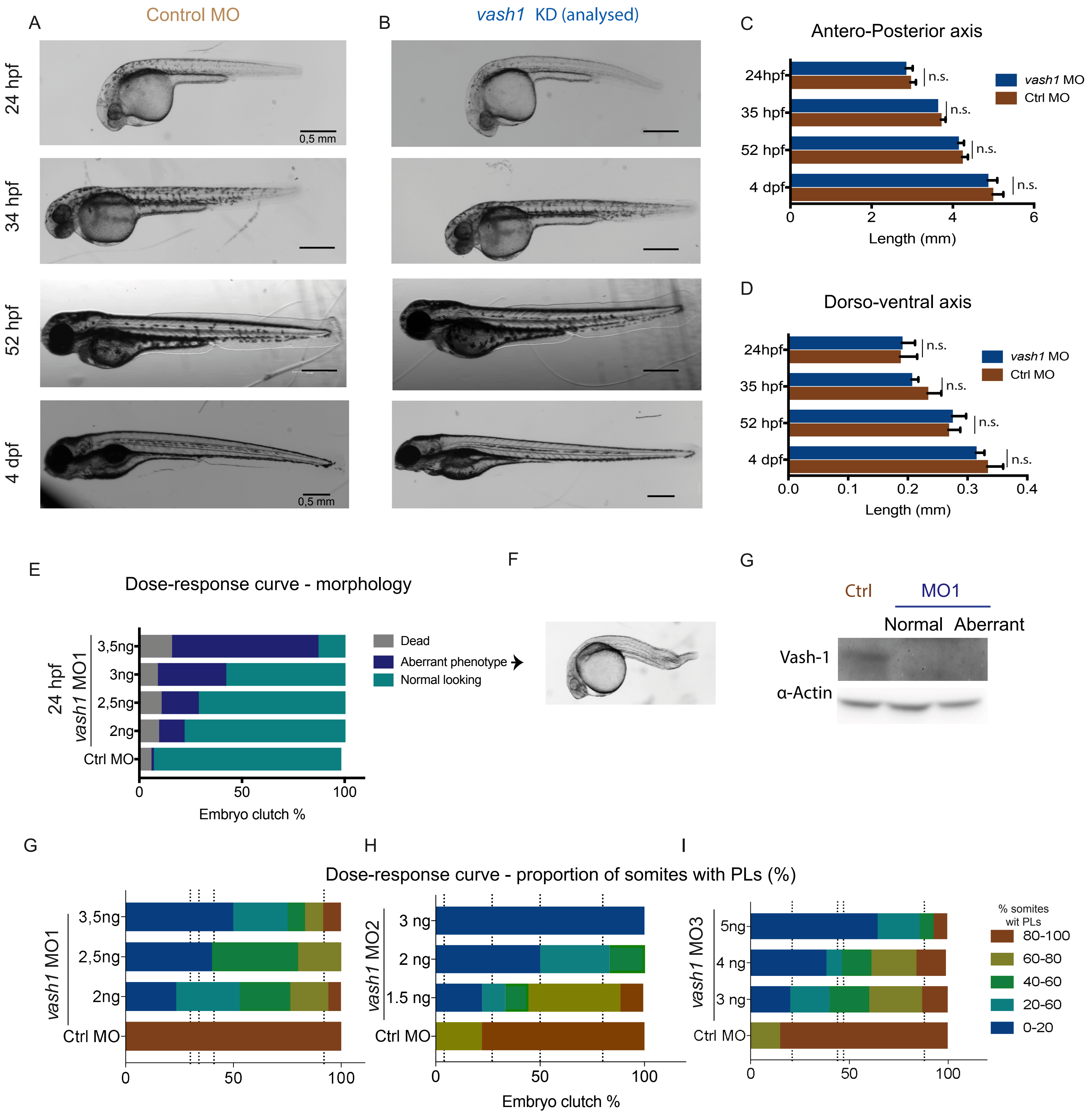

Development • Supplementary information

Figure S1.

Characterisation of development and morphology of *vash1* morphants.

A-D Developmental series of embryos injected with control and *vash1* morpholino at 24, 34, 52 hpf and 4 dpf. Pictures of development of representative and analysed control embryos (A) and *vash1* morphants (B). Progression of growth quantified by antero-posterior (C) and dorso-ventral length (D). **E-G**. Morphological dose response curve of *vash1* morpholino 1 upon injection of 2, 2.5, 3 or 3.5 ng of *vash1* MO 1 (E), generating an increasing proportion of highly morphologically affected wild-type embryos at 24hpf (F) which were not used for further analysis in this project. Normal looking *vash1* morphants exhibit an efficient decrease of Vash1 (G). **H-J** PL dose response curves, determined by percentage of somites with PLs in each *Tg[fli1a:EGFP]*^{y1} embryo injected with control morpholino and specified amounts of *vash1* MO 1 (H), 2 (I) and 3 (J). Dashed lines indicate the average percentage of somites with PLs. Only morphologically normal embryos are analysed in G-H. N= 20/morpholino for dose response curves, from 2 experimental replicates.

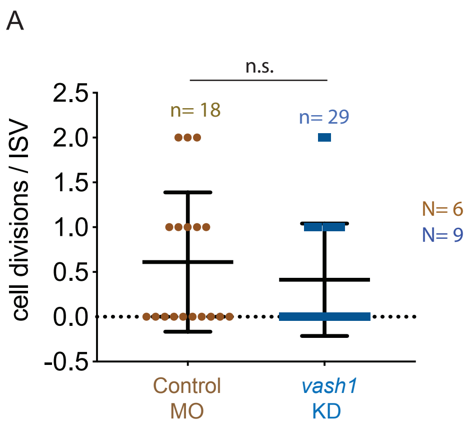

Figure S2.

Vash1 does not affect cell proliferation in the ISVs.

A Cell division events in ISVs of *Tg[kdr-l:ras-Cherry*^{s916}*,fli1a:nEGFP*^{y7}*]* embryos, with membrane- and nuclei-labelled endothelial cells in control and vash1 KD embryos. Quantification obtained from movies from 30 to 70 hpf, by performing Mann-Whitney test. n.s.=non significative.

Development • Supplementary information

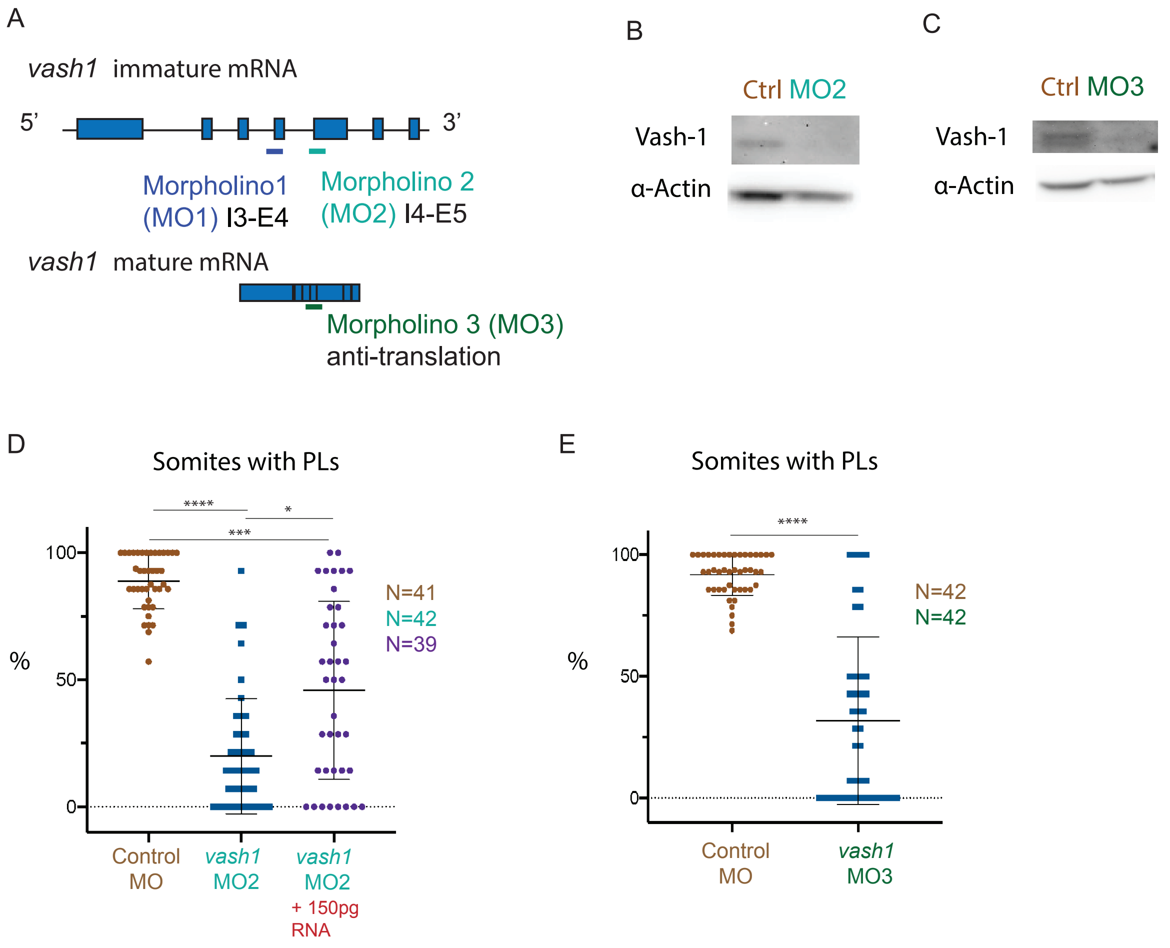

Figure S3.

The morphant phenotype is validated by the use of two additional morpholinos and rescue.

A Validation of the MO1 experiment includes confirmation of the phenotype with independent MOs targeting a different splice region of the immature *vash1* mRNA (MO2) as well as a translation blocking morpholino (MO3). **B-E** MO2 and MO3 efficiently knocked down Vash1 protein levels (B,C) and decreased the PL frequency in the trunk of the morphants (D,E). The MO2 phenotype was rescued by co-injecting *vash1* mRNA (D). N= 41 for controls, N=42 for MO2 injected morphants and N=39 for MO2 and RNA rescue injected embryos. N=44 controls and N=42 for vash1 MO3 injected embryos. For all quantifications, 7-8 somites per embryo were quantified, from 3 biological replicates. p-value was calculated using Kruskal-Wallis (D) and Mann-Whitney (E) test. ****, p<0.0001, ***=<0.0002, * = p<0.0332.

Development • Supplementary information

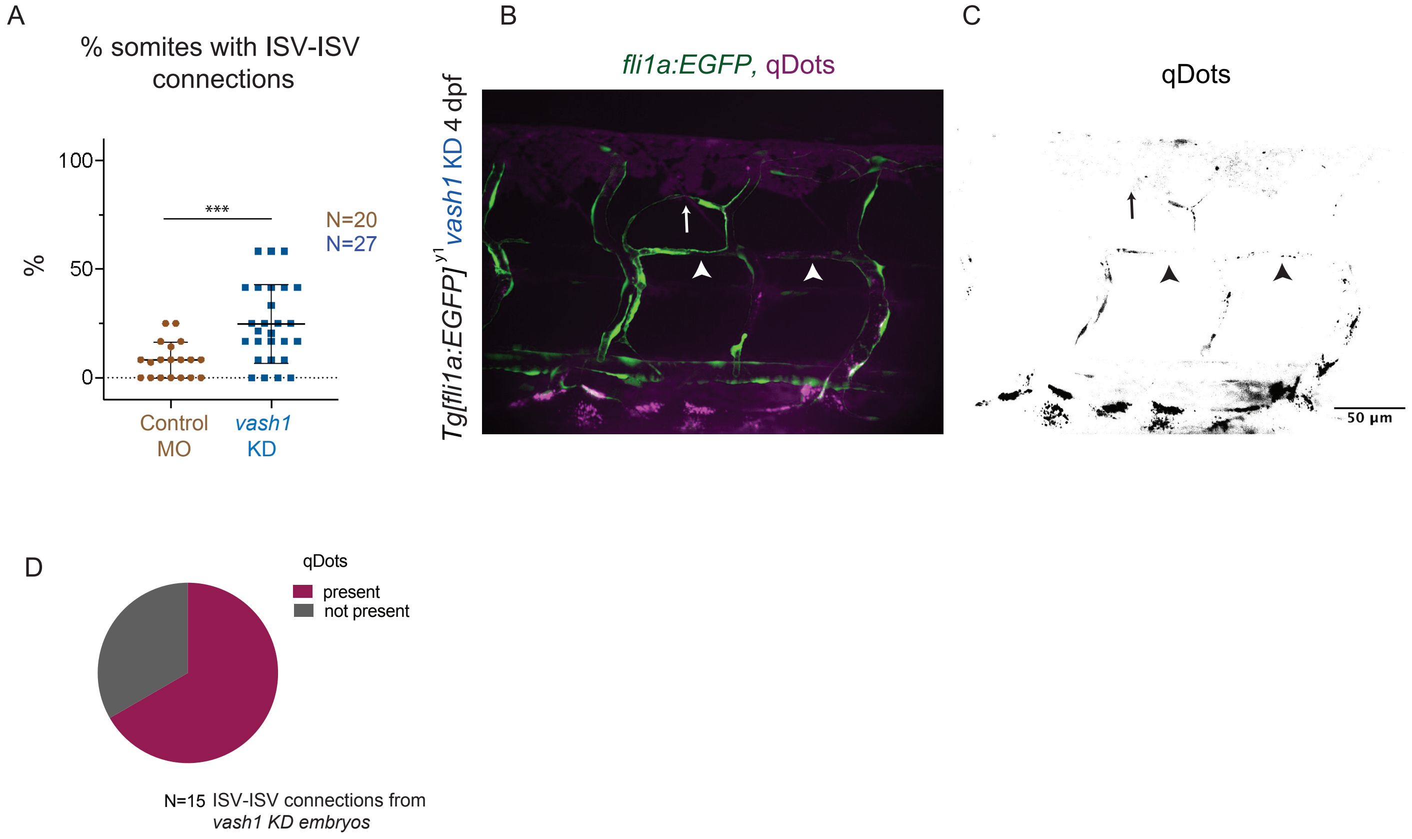

Figure S4.

***vash1* morphants exhibit ISV-ISV connections, of which two thirds are lumenised.**

A About a third (9/27) of 4 dpf *Tg[fli1a:EGFP]^{y1} vash1* morphants exhibit ISV-ISV connections usually absent in control morpholino injected embryos, N=20 for controls and N=27 per *vash1* morphants. p-value was calculated with Mann-Whitney test, ***=<0.0002. **B-D** To assess the serum perfusion of these connections, 4 dpf *vash1* morphants were injected with quantum dots (qDots, in magenta and black in B,C). Arrow points at a non-lumenized connection (B,C), arrow heads point at connections perfused with qDots (B,C). 15 ISV-ISV connections from *vash1* morphants were assessed in respect to presence and absence of qDots (D). Pictures are representative of 3 replicated experiments.

A

Embryo	Mutated clones (in 5)	Indel	Mutation	K174	C175	aa
control	-	-	-	K	C	370
#1	3	9d	in frame deletion	unaffected	C -> A	367
		8d	nonsense mutation	K -> R	C -> G	196
#2	2	2d	nonsense mutation	unaffected	C -> F	289
		1i	nonsense mutation	not translated	not translated	175
#3	4	4d	nonsense mutation	K -> N	C -> W	231
		1i	nonsense mutation	unaffected	C -> L	313
		4s	in frame substitution	K -> N	C -> Q	371
		7d	nonsense mutation	K -> W	C -> R	230
#4	1	7d	nonsense mutation	unaffected	C -> R	229
#5	1	13d	nonsense mutation	K -> L	C -> L	227
#6	2	9i,2d	nonsense mutation	unaffected	C -> F	201
		17d	nonsense mutation	K -> Y	C -> P	194
#7	1	2i,2s	nonsense mutation	unaffected	C -> A	232
#8	3	123d	nonsense mutation	K -> L	C -> P	189
		3d	in frame deletion	K -> S	C -> L	349
		2d	nonsense mutation	unaffected	C -> F	198
#9	2	1i	nonsense mutation	K -> L	C -> F	199
		11i,5d	frameshift mutation	unaffected	C -> I	372
#10	4	2s,4d	nonsense mutation	K -> F	C -> G	197
		6i,14d	nonsense mutation	K -> G	C -> G	196
		3d	frameshift mutation	K -> S	C -> L	369
		13d	nonsense mutation	K -> L	C -> L	227
#11	3	6d	in frame deletion	K -> L	C -> E	369
		22d	nonsense mutation	K -> L	C -> E	224
		1d	nonsense mutation	K -> N	C -> V	231
#12	4	2d	nonsense mutation	unaffected	C -> F	198
		3d	in frame deletion	K -> N	C -> L	369
		3d	in frame deletion	K -> C	C -> L	369
		9i	in frame insertion	unaffected	C -> F	373
13	1	6d	In frame deletion	unaffected	C -> E	
14	2	59i,4d	nonsense mutation	not translated	not translated	184
		39d	In frame deletion	N	S	357

Figure S5.

F0 *vash1* CRISPants exhibit a variety of indels, leading to perturbance of critical amino acids for Vash1 function

A Genotyping of 5 clones per *Tg[fli1a:EGFP]*^{y1} embryo injected with Cas9 and control or dgRNA against exon 4 of *vash1* showed specific mutagenesis in exon 4, not present in the controls, as represented in the first row of the table. CRISPants revealed variation in number of mutated clones (1-4/5) as well as the indels they carry (d-deletion, i- insertion, s-substitution). Often, the mutagenesis caused a premature stop codon, leading to translation of a shorter version of Vash1, as shown in the last table column (control is 370 AA). In all assessed mutants, the cysteine in position 175 of Vash1 was perturbed, impeding the detyrosination of microtubules. Indel stands for insertion-deletion, K174 and C175 stand for lysine and cysteine in positions 174 and 175 respectively, AA stands for amino acid.

Development • Supplementary information

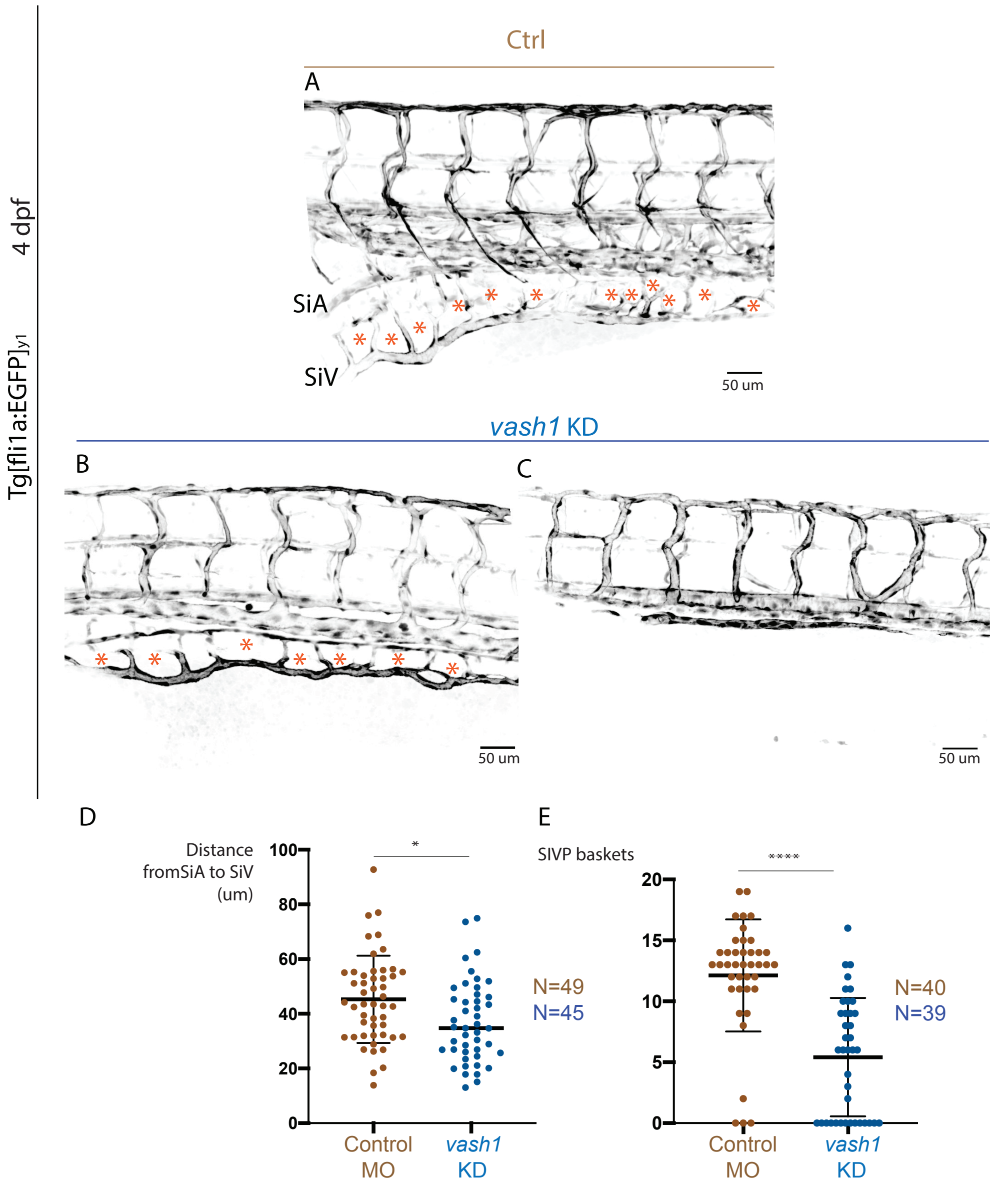

Figure S6. Vash1 deficient embryos show underdeveloped intestinal vascular system.

A-C *Tg[fli1a:EGFP]*^{y1} *embryos* injected with control (A) and *vash1* MO1 (B,C) at 4dpf. SiA, supra-intestinal artery; SiV, supra-intestinal vein. Asterisks label the sub-intestinal venous plexus baskets. Scale is 50um. **D-F** Quantifications of the sub-intestinal venous plexus phenotype in parameters such as the length from the SiA to SiV (D) and number of baskets (E). Three replicates were quantified. p-values calculated with t-test (D) and Mann-Whitney test (E). *p<0.03, ****p<0.0001. Pictures are representative of 3 replicated experiments.

Development • Supplementary information

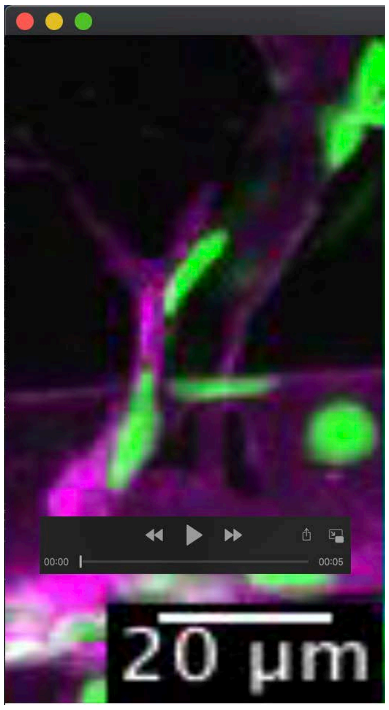

Movie 1. Secondary sprouts in *Tg[kdr-l:ras-Cherrys916,fli1a:nEGFPy7]* embryos, with membrane and nuclei-labelled endothelial cells sprout at around 32-34 hpf. Cells undergoing mitosis are indicated with an arrowhead, as well as their daughter cells. In this movie, a control secondary sprout divides once. Time interval is 15 minutes. Movies are representative of three replicated experiments.

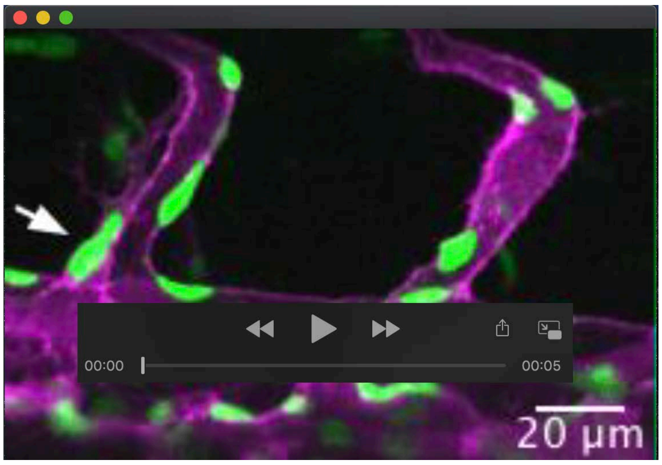

Movie 2. Secondary sprouts in *Tg[kdr-l:ras-Cherrys916,fli1a:nEGFPy7]* embryos, with membrane and nuclei-labelled endothelial cells sprout at around 32-34 hpf. Cells undergoing mitosis are indicated with an arrowhead, as well as their daughter cells. In this movie, a *vash1* KD secondary sprout divides twice. Time interval is 15 minutes. Movies are representative of 3 replicated experiments.

Development • Supplementary information
