# Supplementary material for: Vasohibin 1 selectively regulates secondary sprouting and lymphangiogenesis in the zebrafish trunk
Source: Development. 2021 Feb 19;148(4):dev194993. doi: 10.1242/dev.194993 (PMC7904002; doi:10.1242/dev.194993)
Supplement: Reviewer comments [file develop-148-194993_review_history.pdf]

Vasohibin 1 selectively regulates secondary sprouting and lymphangiogenesis in the zebrafish trunk

Marta Bastos de Oliveira, Katja Meier, Simone Jung, Eireen Bartels-Klein, Baptiste Coxam, Ilse Geudens, Anna Szymborska, Renae Skoczylas, Ines Fechner, Katarzyna Koltowska and Holger Gerhardt
DOI: 10.1242/dev.194993

Editor: Stephen Wilson

Review timeline

Transfer from Review Common received:	15 July 2020
Editorial decision:	30 July 2020
First revision received:	15 December 2020
Accepted:	14 January 2021

Submission to Review Commons

Reviewer 1

Evidence, reproducibility and clarity

The manuscript entitled "Vasohibin-1 mediated tubulin detyrosination selectively regulates secondary sprouting and lymphangiogenesis in the zebrafish trunk" by de Oliveira investigates the function of the carboxylpeptidase Vasohibin during the formation of the zebrafish trunk vasculature and reports a requirement of Vasohibin for secondary sprout formation and in particular the formation the lymphatic vasculature.

Having established the expression of Vasohibin in sorted ECs of 24 hpf embryos, the remaining study addresses the function of Vasohibin in this cell type. It is largely based on the use of a splice-site interfering morpholino. Particular commendable is the analysis, demonstrating that the KD of vash-1 indeed results in a significant reduction of detyrosination in endothelial tubulin. Findings in the vascular system then include: (i) the detection of increased division and hence supernumerous cells occurring selectively in 2nd sprouts from the PCV; (ii) an increased persistence of the initially formed 3 way connections with ISV and artery; (iii) reduced formation of parachordal lymphangioblasts and (iv) a reduced number of somites with a thoracic duct segment; (v) frequent formation of lumenized connections between PLs (where present) and ISV. To demonstrate specificity, the approach was repeated with a different morpholino and defects were partially rescued by MO-insensitive RNA.

Possible additional and relevant information could include data on a vash-1 promotor mutant to independently verify the MO-based functional analysis. Mutants would also allow analysis of further development, are the defects leading to the demise of the fish or is a later regeneration and normalization of the lymphatic vasculature observed? In addition, are other lymphatic vessel beds like the cranial lymphatics affected? PLs have been demonstrated to be at least partially guided in their movement by the CXCR4/SDF1 system and SVEP1. Has the expression of these factors been tested in vash-1 KDs? With regards to the frequently observed connections of PLs and ISVs in vash-1 morphants, can the proposed lumen formation of these shunts be demonstrated e.g. by injection of Q-dots or microbeads into the circulation? Concerning the mechanisms of these defects, is it possible to analyse the asymmetric cell division leading to 2nd sprouts in greater detail? Is the same number or are more cells sprouting form PCV and can the fli1ep:EGFP-DCX cell line in fixed samples be used to identify the spindle orientation in dividing cells?

© 2021. Published by The Company of Biologists under the terms of the Creative Commons Attribution License (https://creativecommons.org/licenses/by/4.0/).

Minor issues:
Page 5, Mat & Meth, please spell out PTU at its first mention.

Page 6 Mat & Meth, Secondary sprout and 3-way connection parameters: The number of nuclei was assessed in each secondary sprouts (del s, singular) just prior...

Page 16, 8th line from bottom: Recent work demonstrated that a secondary sprout either contributes (add s) to remodelling a pre-existing ISV into a vein, or forms (add s)a PLs (Geudens et al., 2019).

Page 25, Legend to Fig. 2D-G: "...G,G' shows quantification of dTyr signal upon vash-1 KD..." Fig2 G,G' show immunostaining rather than quantification of the dTyr signal, which is shown Fig. 2H-J

Fig. 1D / Fig. 2H-J please increase weight of the error intervals and / or change colour for improved visibility

Significance

Taken together the manuscript is comprehensively written and the study provides a conclusive analysis of the MO-mediated KD of Vasohibin in zebrafish embryonic development presenting significant novel findings.
Known was a generally inhibitory function of Vasohibin on vessel formation and its enzymatic activity as a carboxylpeptidase responsible for tubulin detyrosination, affecting spindle function and mitosis. New is the detailed analysis of the Vasohibin KD on zebrafish trunk vessel formation and the description of a selective impairment of 2nd sprout formation.
The manuscript is of interest for vascular biologists.

REFEREES CROSS-COMMENTING

I fully concur with the comments of reviewer #2, all three reviews find that this study is of significant interest to the vascular biology community as the relevance of tubulin detyrosination for developmental angiogenesis has not been investigated. Also all three reviews highlight the potential limitations of the use of splice morpholinos (suggested alternatives include ATG morpholinos and CRIPR mutants), the requirement to provide further evidence for a endothelial cell autonomous defect and the need to clarify some of the data representation.

Reviewer 2

Evidence, reproducibility and clarity

Summary:

The manuscript by Bastos de Oliveira et al. describes an important investigation of the endothelial tubulin detyrosination during vascular development. Namely, they found detyronised microtubules in secondary sprouts, which is absent in MO-vash-1 treated embryos. The authors use the vash-1 morpholino approach to uncover the developmental consequences of suppressed detyrosination in angiogenesis and lymphangiogenesis in vivo in zebrafish. By a combination of transgenic lines, immunohistochemistry and time-lapse imaging, Bastos de Oliveira et al., have found that Vash-1 is a negative regulator of secondary sprouting in zebrafish. The authors showed that in the absence of Vash-1 more cells are present in the secondary sprouts due to increased cell proliferation; however lymphatic vascular network fails to form. The current manuscript requires additional experimental evidence to support the conclusions. Please see below the major technical concerns and minor comments.

Major comments:

-This study is based on analysis of the phenotypes observed in embryos injected with vash-1 morpholino. The authors use two different types of splice morpholinos, perform rescue experiments

© 2021. Published by The Company of Biologists under the terms of the Creative Commons Attribution License (https://creativecommons.org/licenses/by/4.0/).

with RNA, and validate one MO-vash-1 with western blot. Morpholinos are not trivial to work with, and the results are variable hence additional controls need to be included, as following the recommendation put together by the zebrafish community (Stainier et, al., Plos Genetics, 2017). As the severity of the phenotypes comparing MO1 with MO2 is different and MO-vash-1 embryos appear developmentally delayed (Figure 2D-F and 5E-F overall size seem to be affected), additional MO is required, for example, ATG-MO or generation of CRISPR mutant would be favourable. All the morpholino used need to be validated using an antibody, RT-PCR and qPCR. It is essential to carry out the rescue experiments for all the MO used in this study and following the guidelines. Including the dose-response curve, data would be informative.

-In addition to EC, the levels of dTyr are lower in MO-vash-1 in neural tube and neurons spanning the trunk (Figrue 2 D-G'). These have been previously shown to be important for secondary sprouting. Is it possible that the observed phenotypes in the secondary sprouting are due to defects in these neurons?

-Embryo number used in this study appears to be low especially in figure 3G, 5D, 5G, to conclude draw conclusions from these experiments, the number of embryos used should be higher than 20. Figure 4J please specify how many embryos were used.

-The authors hypothesise that VASH acts in the sprouting endothelial cells, based on the Q- PCR in Figure 1. However, in this experiment all EC have been sorted thus this remains ambiguous in which cell types vash-1 is expressed. Please provide the expression pattern for vash-1 across the developmental stages the phenotypes are observed.

-Throughout the manuscript the authors refer the lymphatic identity, however, there is no evidence in the paper that the identity status has been assessed. To support these claims Prox1 immunohistochemistry or analysis of prox1 expression in the reporter line would be appropriate.

Minor comments:

-The authors refer to the literature where overexpression of VASH suppresses the angiogenesis. As the RNA injections were used in rescue experiments, the data of vash-1 RNA injections into the wild-type embryos would be beneficial.

-In figures 2J, 3J, 3K, 3N, 4J, 5C, 5D and 5G the N number was set for examples as the number of sprouts, the number of somites with TD, number of ISV. To strengthen the observation in the manuscript quantification of the sprouts, PL, vISVs and lymphatic phenotypes with N set as the number of embryos would be more informative. Indicating the number of embryos used, in the graphs, would be helpful.

-In Figure 5A, B and D the authors quantify what they refer to as a lumenised connection between the vISVs and PL. In the control image (second star), a somewhat lumenised structure is present, clarification of how the scores were set is missing.

-In Figure 3 E and F the authors show the excessive sprouting phenotype between controls and Mo-vash-1. The images presented are taking from different parts of the embryos (middle of the trunk vs plexus region), hampering the comparison between the two groups. The quantification of the phenotypes in both experimental groups should be in the same region of the embryo, as the local difference can occur. It is key to provide representative images to support these observations.

-Figure 1D the vash-1 expression levels in EC seem very variable in this graph, therefore no conclusion can be drawn from this data, especially as the authors do not provide the p- values.

-In the introduction, the authors state: 'Although primary and secondary sprouts appear morphologically similar, with tip and stalk cells' - Please provide the reference that supports the claim that secondary sprouts have tip-stalk cells morphology/organisation.

-The authors refer the increased cell division phenotypes observed in the movies, however, the movie files have not been available to the reviewers.

© 2021. Published by The Company of Biologists under the terms of the Creative Commons Attribution License (https://creativecommons.org/licenses/by/4.0/).

Significance

This is an important study as uncovering the mechanistic details of angiogenic and lymphangiogenic negative regulators is of high value with the potential for therapeutic developments. To date, Vash-1 has been only studied in the context of tumour angiogenesis, vasculature in diabetic nephropathy and pulmonary arterial hypertension, and it remains unclear what is its role during development and how does it regulate vascular network formation. The tyrosination status of microtubule in endothelial cells is understudied. This study revealed, previously uncharacterised detyrosinated microtubules in endothelial cells in vivo. And further dissects how this process might be regulated, brings unique insights into the vascular biology field and beyond. Thus, delving into the cell biological mechanism such as microtubule dynamics and modification in vivo in embryo context is a significant step forward in setting new standards in the field.

I am developmental biologist who has experience in model organisms such as zebrafish and mouse. The main focus of my work is on developmental angiogenesis and lymphangiogenesis.

REFEREES CROSS-COMMENTING

After reading the other reviews comments, it seems that we all agree that this study is of high value to vascular biology field and beyond bringing novel findings.

Importantly the reviewers' comments are in line with each other and have identified several commonalities that should be addressed. Such as:
Further validation of Morpholinos, or using alternative methods to replicate the findings.
additional evidence that the observed phenotypes are primary due to vash-1 requirement within EC, and not due to the secondary effect in other cells such as CXCR4/SDF1 system and SVEP1, neurons or general delay of the embryos
Further evidence of for VASH expression pattern
the number of embryos used in the experiments, and how the data is represented.

Reviewer 3

Evidence, reproducibility and clarity

Vasohibin-1 (Vash-1) is known to detyrosinate microtubules (MTs) and limit angiogenesis.
Using in vivo live imaging and whole mount immunofluorescence staining of zebrafish trunk vasculature, Bastos de Oliveira et al. show that the MT detyrosination role of Vash-1 is conserved in zebrafish and that Vash-1 is essential for limiting venous sprouting and subsequent formation of lymphatics. Their findings suggest a role for MT detyrosination in lympho-venous cell specification.

Major comments:

1 . The authors claim that Vash-1 regulates secondary sprouting and lymphangiogenesis by detyrosinating MTs. However, no direct evidence of this link is provided in the manuscript.
The authors only separately show that knockdown of vash-1 affects MT detyrosination and secondary sprouting and lymphangiogenesis. They have not shown a causative effect. The authors should therefore qualify the above stated claim as speculative. In other words, the authors should mention that their data only suggests that disruption of MT detyrosination is the underlying cause for aberrant secondary sprouting and lymphangiogenesis in vash-1 KD embryos.

2 . In order to provide more compelling evidence for a direct relationship between MT tyrosination and lymphangiogenesis, the authors could try mutating the carboxypeptidase domain of vash-1 or overexpressing a dominant negative transcript (that contains a mutated carboxypeptidase domain). If this gives the same phenotypes as the vash-1 morphants, it would indicate that the carboxypeptidase activity of Vash-1 (in detyrosinating MTs) is responsible for limiting secondary sprouting and promoting specification of lymphatics. This suggested experiment is fairly realistic in terms of both time and resources. For example, since the authors already have the human vash-1 cDNA cloned, making a dominant negative transcript from this would take around two weeks,

© 2021. Published by The Company of Biologists under the terms of the Creative Commons Attribution License (https://creativecommons.org/licenses/by/4.0/).

imaging and analysis of embryos injected with this mRNA would take another four weeks. Therefore, in total, the suggested experiment would take around 6 weeks. Although the alternative experiment, that is, making a carboxypeptidase domain mutant of vash-1 would be a better choice in terms of reproducibility and long-term use of a stable line, it would admittedly take a relatively larger amount of time. Therefore, the ultimate choice would depend on the authors.

3 . Both the data and methods are presented in a way that ensures reproducibility. The statistical analysis is very well done, in that the authors were very prudent in their choice of statistical tests. However, in many figures and subfigures (Fig. 2B, H-J; Fig. 3G, J, K, N; Fig. 4J; Fig. 5J), the number of replicates was not mentioned and instead only the sample size was stated. Whether this was just an oversight or if it should be taken to mean that the analysis was performed on just one replicate is unclear. The authors need to clarify this aspect of their analysis. Further, In Fig. 2H-J, Fig. 3G,J, K, N and Fig. 4J, the total number of data points in control MO vs vash-1 KD seem to be quite different. In other words, there seem to be a lot more data points in one experimental condition than the other. Does this difference fall within the acceptable range? If the authors were to compare a similar number of data points between the two experimental conditions, would the results of the statistical analysis still be the same?

4 . The authors only provide KD data on the function of vash-1 using morpholinos. According to several recent guidelines concerning the use of morpholinos, this is not widely accepted in the zebrafish community as sufficient to provide robust insight into gene function. Please refer for example to the following publication: Guidelines for morpholino use in zebrafish, Stainier et al., PLOS Genetics, 2017. The generation of a vash-1 mutant is a necessary requirement for backing up morpholino KD data. Further, even though the authors state that embryos were selected on the pre-established criteria that they have normal morphology, beating heart, and flowing blood, certain morphological differences between control MO injected and vash-1 KD embryos could be observed in some figures. In Fig. 2D, F and Fig. 5A, B, E, F the vash-1 KD embryos seem smaller (extend of the dorso-ventral axis) than control MO injected embryos. The authors need to provide images showing the overall morphology of morpholino injected embryos and need to provide evidence that morpholino injections do not cause developmental delays.

Minor comments:

a. The authors should back their qPCR data for vash-1 expression (Figure 1) by standard mRNA in situ hybridization, given the large degree of variability in vash-1 expression. Do they observe a dynamic expression in the vasculature using this technique?

b. The number of nuclei per sprout in Fig. 3J does not correspond with the number of divisions per sprout presented in Fig. 3K. The authors observe one or two cell divisions per sprout in ctr MO injected embryos (Fig. 3K), however, Fig. 3J shows that the majority of ctr. sprouts contains only one cell. This is even more dramatic for vash-1 MO injected embryos, which can have up to four divisions, therefore should contain six cells. However, the maximum number of cells the authors report is three to four cells. How do these observations go together?

c. Fig. 5I and J have the same data points for control MO and vash-1 MO1. Does this mean that both graphs are from the same experiment? If so, the authors could combine the two graphs into one. If the two graphs are not from the same experiment, both would need to have independent controls.

d. The percentage of somites with PLs in vash-1 MO1 injected embryos in Fig. 5I is half the value shown in Fig. 5C. Although this kind of variability might be expected in biological samples, perhaps the authors could briefly discuss the issue and its implications on reproducibility in the manuscript so as to have the readers be aware of it, especially since the rescue of the vash-1 morpholino phenotype back to 50% from 25% is the same value the authors observed in the vash-1 KD alone in Fig. 5C. Here the value is 50% for the morpholino injection.

e. The Y-axis label is missing in Fig. 2H and Fig. 4J. Figure 5D lacks bars showing median and standard deviation.

f. It would help to have an inference or conclusion at the end of each results section.

© 2021. Published by The Company of Biologists under the terms of the Creative Commons Attribution License (https://creativecommons.org/licenses/by/4.0/).

Significance

Conceptual: As per my knowledge, this is the first study that looks at microtubule modifications in the context of a vertebrate organism past the gastrulation stage, as opposed to similar studies that have been done in cell culture or invertebrates (S. cerevisiae, C. elegans and D. melanogaster). Moreover, this study is one of few that address a novel link between the cytoskeleton and the process of cell fate specification.

Previous studies have separately shown that Vash-1 limits angiogenesis and detyrosinates MTs. The current study combines the two observations in the context of endothelial cells, and hypothesizes that perhaps the function of Vash-1 in limiting angiogenesis and at the same time promoting lymphatic development could be due to its role in MT modification at the molecular level and the consequent effect of this on cell division and/or fate specification at the cellular level. In short, this study aims to connect the long-standing gap in knowledge between cytoskeletal modifications and cell dynamics (in particular, division and specification) in a vertebrate organism. I therefore believe that the current study would be an exciting finding for research communities that study cytoskeletal influence on cellular dynamics and also those in the broad area of vascular biology.

My field of expertise relates to vascular biology, specifically developmental angiogenesis and the behavior of endothelial cells in zebrafish.

Author response to reviewers' comments

Reviewer #1 (Evidence, reproducibility and clarity (Required)):

The manuscript entitled "Vasohibin-1 mediated tubulin detyrosination selectively regulates secondary sprouting and lymphangiogenesis in the zebrafish trunk" by de Oliveira investigates the function of the carboxylpeptidase Vasohibin during the formation of the zebrafish trunk vasculature and reports a requirement of Vasohibin for secondary sprout formation and in particular the formation the lymphatic vasculature.

Having established the expression of Vasohibin in sorted ECs of 24 hpf embryos, the remaining study addresses the function of Vasohibin in this cell type. It is largely based on the use of a splice-site interfering morpholino. Particular commendable is the analysis, demonstrating that the KD of *vash-1* indeed results in a significant reduction of detyrosination in endothelial tubulin. Findings in the vascular system then include:
(i) the detection of increased division and hence supernumerous cells occurring selectively in 2nd sprouts from the PCV; (ii) an increased persistence of the initially formed 3 way connections with ISV and artery; (iii) reduced formation of parachordal lymphangioblasts and (iv) a reduced number of somites with a thoracic duct segment; (v) frequent formation of lumenized connections between PLs (where present) and ISV. To demonstrate specificity, the approach was repeated with a different morpholino and defects were partially rescued by MO-insensitive RNA.

Possible additional and relevant information could include data on a *vash-1* promotor mutant to independently verify the MO-based functional analysis. Mutants would also allow analysis of further development, are the defects leading to the demise of the fish or is a later regeneration and normalization of the lymphatic vasculature observed?

We agree that a mutant would be desirable to validate the phenotypic analysis of the morpholinos used, and would also allow for further analysis. However, this is not achievable within a reasonnable time frame, especially in the context of current work restrictions.
In addtion to the two splice morpholinos currently used to knockdown *vash-1* expression, we will use an ATG morpholino to further investigate our observations and hypothesis regarding

© 2021. Published by The Company of Biologists under the terms of the Creative Commons Attribution License (https://creativecommons.org/licenses/by/4.0/).

the role of *vash-1* in lymphatic vessels formation. We will also validate it by westernblot and attempt to rescue it with mRNA.

We have not investigated the phenotype past 4 dpf. We will add investigation of lymphatics and morphology at 5 dpf.

In addition, are other lymphatic vessel beds like the cranial lymphatics affected?

Using the *Tg[fli1a:EGFP]^{y7}* line, we have not been able to identify apparent differences in other vascular beds including the cranial lymphatics. However a detailed fine-grained investigation of the cranial vascular bed has not been performed. Given the focus of the present study on the trunk vasculature to understand the mechanisms of vash-1, we feel that a detailed analysis of cranial lymphatics would at this stage be somewhat out of scope.

PLs have been demonstrated to be at least partially guided in their movement by the CXCR4/SDF1 system and SVEP1. Has the expression of these factors been tested in *vash-1* KDs?

We have not investigated the potential role of the CXCR4/SDF1 system and SVEP1 in *vash-1* regulation of lymphangiogenesis. We will investigate the expression of *cxcr4a, cxcl12a, cxcl12b* and *svep1* by *in situ* hibridization upon *vash-1* knockdown.

With regards to the frequently observed connections of PLs and ISVs in *vash-1* morphants, can the proposed lumen formation of these shunts be demonstrated e.g. by injection of Q-dots or microbeads into the circulation?

Although the lumenisation is very clear thanks to the membrane targeted expression of the label in this line, we will further analyse whether these abberant ISV to ISV connection can be perfused by Q-dots injections.

Concerning the mechanisms of these defects, is it possible to analyse the asymmetric cell division leading to 2nd sprouts in greater detail? Is the same number or are more cells sprouting form PCV and can the fli1ep:EGFP-DCX cell line in fixed samples be used to identify the spindle orientation in dividing cells?

We agree with the reviewer and plan to use the *Tg[fli1ep:EGFP-DCX]* fish line to investigate spindle asymmetry in uninjected embryos, as well as compare the spindle in control MO and *vash-1* KD embryos. Vash-1 has been shown to regulate spindle formation in osteosarcoma cells (Liao et al., 2019). We will attempt to clarify whether this function is conserved in endothelial cells and contributes to the control of endothelial cell proliferation during initiation and formation of secondary sprouting.
We also agree that it is important to look at the PCV in the begining of secondary sprouting and will clarify whether the sprouting is initiated by an increased number of cells.

Minor issues:
Page 5, Mat & Meth, please spell out PTU at its first mention.

This has been corrected accordingly (see page 4).

Page 6 Mat & Meth, Secondary sprout and 3-way connection parameters: The number of nuclei was assessed in each secondary sprouts (del s, singular) just prior...

This has been corrected accordingly (see page 5).

Page 16, 8th line from bottom: Recent work demonstrated that a secondary sprout either contributes (add
s) to remodelling a pre-existing ISV into a vein, or forms (add s)a PLs (Geudens et al., 2019).

This has been corrected accordingly (see page 16).

Page 25, Legend to Fig. 2D-G: "...G,G' shows quantification of dTyr signal upon *vash-1* KD..."

© 2021. Published by The Company of Biologists under the terms of the Creative Commons Attribution License (https://creativecommons.org/licenses/by/4.0/).

Fig2 G,G' show immunostaining rather than quantification of the dTyr signal, which is shown Fig. 2H-J

This has been corrected accordingly (see page 26).

Fig. 1D / Fig. 2H-J please increase weight of the error intervals and / or change colour for improved visibility

This has been corrected accordingly (Fig. 1D and 2H-J), and we added n.s. to Fig. 1D.

Reviewer #1 (Significance (Required)):

Taken together the manuscript is comprehensively written and the study provides a conclusive analysis of the MO-mediated KD of Vasohibin in zebrafish embryonic development presenting significant novel findings.

Known was a generally inhibitory function of Vasohibin on vessel formation and its enzymatic activity as a carboxylpeptidase responsible for tubulin detyrosination, affecting spindle function and mitosis. New is the detailed analysis of the Vasohibin KD on zebrafish trunk vessel formation and the description of a selective impairment of 2nd sprout formation.
The manuscript is of interest for vascular biologists.

REFEREES CROSS-COMMENTING

I fully concur with the comments of reviewer #2, all three reviews find that this study is of significant interest to the vascular biology community as the relevance of tubulin detyrosination for developmental angiogenesis has not been investigated. Also all three reviews highlight the potential limitations of the use of splice morpholinos (suggested alternatives include ATG morpholinos and CRIPR mutants), the requirement to provide further evidence for a endothelial cell autonomous defect and the need to clarify some of the data representation.

Reviewer #2 (Evidence, reproducibility and clarity (Required)):

Summary:

The manuscript by Bastos de Oliveira et al. describes an important investigation of the endothelial tubulin detyrosination during vascular development. Namely, they found detyronised microtubules in secondary sprouts, which is absent in MO-vash-1 treated embryos. The authors use the vash-1 morpholino approach to uncover the developmental consequences of suppressed detyrosination in angiogenesis and lymphangiogenesis in vivo in zebrafish. By a combination of transgenic lines, immunohistochemistry and time-lapse imaging, Bastos de Oliveira et al., have found that Vash-1 is a negative regulator of secondary sprouting in zebrafish. The authors showed that in the absence of Vash-1 more cells are present in the secondary sprouts due to increased cell proliferation; however lymphatic vascular network fails to form. The current manuscript requires additional experimental evidence to support the conclusions. Please see below the major technical concerns and minor comments.

Major comments:

-This study is based on analysis of the phenotypes observed in embryos injected with vash-1 morpholino. The authors use two different types of splice morpholinos, perform rescue experiments with RNA, and validate one MO-vash-1 with western blot. Morpholinos are not trivial to work with, and the results are variable hence additional controls need to be included, as following the recommendation put together by the zebrafish community (Stainier et, al., Plos Genetics, 2017). As the severity of the phenotypes comparing MO1 with MO2 is different and MO-vash-1 embryos appear developmentally delayed (Figure 2D-F and 5E-F overall size seem to be affected), additional MO is required, for example, ATG-MO or generation of CRISPR mutant would be favourable. All the morpholino used need to be

© 2021. Published by The Company of Biologists under the terms of the Creative Commons Attribution License (https://creativecommons.org/licenses/by/4.0/).

validated using an antibody, RT-PCR and qPCR. It is essential to carry out the rescue experiments for all the MO used in this study and following the guidelines. Including the dose-response curve, data would be informative.

We agree with the reviewer and the recommendations of the zebrafish community. We will investigate the phenotypes with another KD strategy, such as the ATG-Morpholino suggested by the reviewer. We will also supply more validation of the MO2 including RNA rescue and westernblot (already included in Fig. 5 I).
We added dose-response curves (Supp. Figure 1 E,G) and a developmental morphology assessment for the morpholino 1 (Supp. Figure 1 A,B).
Given our extensive analysis of the effects of *vash-1* KD, we believe the embryos in 2F are not developmentally delayed. However, the image in figure 2F does give that impression, and therefore may have triggered the reviewer's concerns. We double checked and found that due to an oversight, we included a picture from a slightly different region of the trunk in comparision to Fig. 2D. We will add pictures of the same trunk region (Fig.2D-F) as we have done in all other figures. We nonetheless supply a supplementary figure 1 showing and quantifying the development of the analysed *vash-1* morphants.

-In addition to EC, the levels of dTyr are lower in MO-vash-1 in neural tube and neurons spanning the trunk (Figrue 2 D-G'). These have been previously shown to be important for secondary sprouting. Is it possible that the observed phenotypes in the secondary sprouting are due to defects in these neurons?

We agree with the reviewer that a potential contribution of altered neuronal differentiation to the vascular phenotype should be clarified.
We will assess the morphology of the neurons and their dendrites relevant for pathfinding (Lim et al., 2011) in *vash-1* KD embryos, using a pan-neuronal zebrafish line, as well as via immunostaining against alpha- tubulin. Should we find evidence for changes in neuronal cells, we will attempt to clarify a cell autonomous role of *vash-1* by transplantation experiments.

-Embryo number used in this study appears to be low especially in figure 3G, 5D, 5G, to conclude draw conclusions from these experiments, the number of embryos used should be higher than 20. Figure 4J please specify how many embryos were used.

We will increase the number of embryos per condition to a minimum of 20 embryos and update the averages in the text for 3G (control: 7 and *vash-1* KD: 11 embryos).

In 5D and 5G each point is an embryo and more than 20 embryos per condition were used (in 5D 23-35 embryos per condition, in 5G 60-63 embryos/condition), we corrected the legend 5D and 5G (see page 27) and made it clear that each point in the graph corresponds to one embryo (5D- percentage of PLs associated with veins in each embryo; 5G- percentage of somites with toraxic duct in each embryo).
In 4J, 18 embryos were used for control (about 3 sprouts/embryo- 52 sprouts quantified) and 7 embryos for *vash-1* KD condition (about 3 sprouts/embryo - 24 sprouts quantified). We corrected the number of control sprouts in the legend and added the number of embryos to increase clarity (see page 27).

-The authors hypothesise that VASH acts in the sprouting endothelial cells, based on the Q-PCR in Figure 1. However, in this experiment all EC have been sorted thus this remains ambiguous in which cell types vash-1 is expressed. Please provide the expression pattern for vash-1 across the developmental stages the phenotypes are observed.

We agree with the reviewer that it would be beneficial to understand the expression pattern of *vash-1* in wild type embryos. We plan to perform *in situ* hybridization for *vash-1* mRNA.

-Throughout the manuscript the authors refer the lymphatic identity, however, there is no evidence in the paper that the identity status has been assessed. To support these claims Prox1 immunohistochemistry or analysis of prox1 expression in the reporter line would be appropriate.

We agree with the reviewer and plan to perform a Prox1 immunostaining (Koltowska et al.,

© 2021. Published by The Company of Biologists under the terms of the Creative Commons Attribution License (https://creativecommons.org/licenses/by/4.0/).

2015) in *vash-1* KD embryos at 34-36 hpf (secondary sprouting) to investigate Prox1 levels upon *vash-1* KD.

Minor comments:

-The authors refer to the literature where overexpression of VASH suppresses the angiogenesis. As the RNA injections were used in rescue experiments, the data of vash-1 RNA injections into the wild-type embryos would be beneficial.

We have injected *vash-1* RNA into a control morpholino injected embryos (28 control embryos, 14 Vash-1 RNA injected embryos) and we observed a significant decrease in PLs at 52 hpf (average of -control: 87,5% somites with PLs to 67% somites with PLs in *vash-1* RNA embryos). This could be due to a decrease of secondary sprouting, which would be in accordance with the current literature that *vash-1* overexpression is anti-angiogenic. We will further investigate and add the results to figure 5.

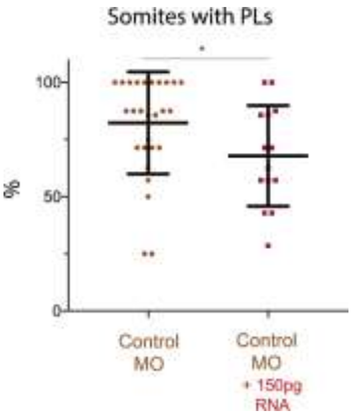

Figure 1. *vash-1* mRNA injection leads to a decrease in somites with PLs (preliminary).

-In figures 2J, 3J, 3K, 3N, 4J, 5C, 5D and 5G the N number was set for examples as the number of sprouts, the number of somites with TD, number of ISV. To strengthen the observation in the manuscript quantification of the sprouts, PL, vISVs and lymphatic phenotypes with N set as the number of embryos would be more informative. Indicating the number of embryos used, in the graphs, would be helpful.

We agree with the reviewer and have added embryo numbers in all legends and graphs. In 2J, 3J, 3K, 4J each point is a sprout, a cell division or an ISV, corresponding to the N. We agree that the number of embryos could be more clearly stated, so we added the number of embryos analysed in the figure legend and will add them in the graphs.

In 5C, 5D and 5G each point corresponds to an embryo (clarified in the legend of Fig. 5- see page 27).
Fig. 5C refers to the percentage of somites with PLs in each embryo, 5D refers to percentage of the existing PLs in one embryo connected to a venous ISV, 5G corresponds to percentage of somites with a TD segment in each embryo.

-In Figure 5A, B and D the authors quantify what they refer to as a lumenised connection between the vISVs and PL. In the control image (second star), a somewhat lumenised structure is present, clarification of how the scores were set is missing.

In Fig. 5C we show a quantification of the percentage of somites with PLs per embryo, by counting the PLs identified with an asterisk in Fig. 5A-B. PLs are normally not lumenised, with few exceptions also ocurring in wild-type - see Fig. 4 in (S Isogai et al., 2001).

In Fig. 5D we quantified the proportion of PLs associated/connected with venous ISvs (see Methods section page 6), by 52 hpf in control and *vash-1* morphants.

© 2021. Published by The Company of Biologists under the terms of the Creative Commons Attribution License (https://creativecommons.org/licenses/by/4.0/).

In 5B and 5F,F‘, the arrowheads identify lumenised PLs present in *vash-1* KD embryos. We will add a quantification of kdr-l:ras-Cherry positive ISV-to-ISV connections, corresponding to the lumenised endothelial connections, since kdr-l:ras-Cherry signal labels endothelial (and not lymphatic) cells and is particularly strong at the luminal endothelial membrane of the vessel.

-In Figure 3 E and F the authors show the excessive sprouting phenotype between controls and Mo-vash-1. The images presented are taking from different parts of the embryos (middle of the trunk vs plexus region), hampering the comparison between the two groups. The quantification of the phenotypes in both experimental groups should be in the same region of the embryo, as the local difference can occur. It is key to provide representative images to support these observations.

The images presented are representative of the phenotype quantified, and the time-lapses were done in comparable regions of the zebrafish trunk (+- 1-2 somites in both groups due to drift during image aquisition), making the comparison possible.

-Figure 1D the vash-1 expression levels in EC seem very variable in this graph, therefore no conclusion can be drawn from this data, especially as the authors do not provide the p-values.

We added n.s. in the graph, to make it clear that the difference between developmental stages is not significant, potentially due to high biological variability between embryos, as seen in two primer pairs. We believe that presenting this biological variability is of importance to the readers.

We write on page 12 about this result: „During the sprouting phase (24hpf), *vash-1* expression was 5-7 times higher in endothelial than in non-ECs, decreasing at 48 hpf (Fig. 1C-D). Although these results are not significant, they were independently confirmed with a second primer set.”. The only conclusion we made from this data is that Vash-1 is dynamically expressed in the zebrafish endothelium during development, as we now added in the discussion (page 14).

-In the introduction, the authors state: 'Although primary and secondary sprouts appear morphologically similar, with tip and stalk cells' - Please provide the reference that supports the claim that secondary sprouts have tip-stalk cells morphology/organisation.

Although many studies have investigated primary and secondary sprouting, identifying both shared as well as distinct molecular regulation, and show morphological details that are apparently similar, a formal claim that secondary sprouts show tip and stalk cell identities and behaviour is hard to find. Given that this is not relevant for the central findings of the work, we modified the sentence and added a reference “Although primary and secondary sprouts appear morphologically similar, ~~with tip and stalk cells~~” (Sumio Isogai et al., 2003)…” See page 2.
We also updated the discussion for consistency: “Although the cellular mechanisms of primary and secondary sprouting in zebrafish appear very similar, ~~with tip cell selection and guided migration and stalk cell proliferation,~~ secondary sprouting utilises alternative signalling pathways and entails a unique specification step that establishes both venous ISVs and lymphatic structures.” (see page 15)

-The authors refer the increased cell division phenotypes observed in the movies, however, the movie files have not been available to the reviewers.

We will provide the movies.

Reviewer #2 (Significance (Required)):

This is an important study as uncovering the mechanistic details of angiogenic and lymphangiogenic negative regulators is of high value with the potential for therapeutic developments. To date, Vash-1 has been only studied in the context of tumour angiogenesis, vasculature in diabetic nephropathy and pulmonary arterial hypertension, and it remains unclear what is its role during development and how does it regulate vascular network formation. The tyrosination status of microtubule in endothelial cells is understudied. This

© 2021. Published by The Company of Biologists under the terms of the Creative Commons Attribution License (https://creativecommons.org/licenses/by/4.0/).

study revealed, previously uncharacterised detyrosinated microtubules in endothelial cells in vivo. And further dissects how this process might be regulated, brings unique insights into the vascular biology field and beyond. Thus, delving into the cell biological mechanism such as microtubule dynamics and modification in vivo in embryo context is a significant step forward in setting new standards in the field.

I am developmental biologist who has experience in model organisms such as zebrafish and mouse. The main focus of my work is on developmental angiogenesis and lymphangiogenesis.

REFEREES CROSS-COMMENTING

After reading the other reviews comments, it seems that we all agree that this study is of high value to vascular biology field and beyond bringing novel findings.

Importantly the reviewers' comments are in line with each other and have identified several commonalities that should be addressed. Such as:
Further validation of Morpholinos, or using alternative methods to replicate the findings.
additional evidence that the observed phenotypes are primary due to vash-1 requirement within EC, and not due to the secondary effect in other cells such as CXCR4/SDF1 system and SVEP1, neurons or general delay of the embryos
Further evidence of for VASH expression pattern the number of embryos used in the experiments, and how the data is represented.

Reviewer #3 (Evidence, reproducibility and clarity (Required)):

Vasohibin-1 (Vash-1) is known to detyrosinate microtubules (MTs) and limit angiogenesis. Using in vivo live imaging and whole mount immunofluorescence staining of zebrafish trunk vasculature, Bastos de Oliveira et al. show that the MT detyrosination role of Vash-1 is conserved in zebrafish and that Vash-1 is essential for limiting venous sprouting and subsequent formation of lymphatics. Their findings suggest a role for MT detyrosination in lympho-venous cell specification.

Major comments:

1 . The authors claim that Vash-1 regulates secondary sprouting and lymphangiogenesis by detyrosinating MTs. However, no direct evidence of this link is provided in the manuscript. The authors only separately show that knockdown of vash-1 affects MT detyrosination and secondary sprouting and lymphangiogenesis. They have not shown a causative effect. The authors should therefore qualify the above stated claim as speculative. In other words, the authors should mention that their data only suggests that disruption of MT detyrosination is the underlying cause for aberrant secondary sprouting and lymphangiogenesis in vash-1 KD embryos.

We agree with the reviewer about the lack of evidence to state that the disruption of microtubule detyrosination leads to aberrant secondary sprouting. Although we believe this is the most parsimonius explanation for the secondary sprouts behavioural defects as cell division is disturbed and microtubule detyrosination is implicated in cell division (Barisic et al., 2015), we want to make clear that our data currently only suggest a specific role of microtubule detyrosination in secondary sprouting. Examples of this are page 14 of the discussion „These results suggest that Vash-1-driven microtubule detyrosination limits excessive venous EC sprouting and proliferation during lympho-venous development in zebrafish." as well as the abstract.

We also corrected the sentence in the discussion (page 14): "In this study, we identified Vash-1~~-mediated microtubule detyrosination~~ as ~~a cellular mechanism~~ as a novel regulator of EC sprouting from the PCV and the subsequent formation of lymphatic vessels in the zebrafish trunk."
To avoid any overstatement, we also propose the following title change: Vasohibin-1 ~~mediated tubulin detyrosination~~ selectively regulates secondary sprouting and lymphangiogenesis in the zebrafish trunk.

© 2021. Published by The Company of Biologists under the terms of the Creative Commons Attribution License (https://creativecommons.org/licenses/by/4.0/).

As detailed in response to comment 2 below, we will however attempt to investigate the direct connection. Depending on the outcome, we will adapt conclusions and title accordingly.

2 . In order to provide more compelling evidence for a direct relationship between MT tyrosination and lymphangiogenesis, the authors could try mutating the carboxypeptidase domain of vash-1 or overexpressing a dominant negative transcript (that contains a mutated carboxypeptidase domain). If this gives the same phenotypes as the vash-1 morphants, it would indicate that the carboxypeptidase activity of Vash-1 (in detyrosinating MTs) is responsible for limiting secondary sprouting and promoting specification of lymphatics. This suggested experiment is fairly realistic in terms of both time and resources. For example, since the authors already have the human vash-1 cDNA cloned, making a dominant negative transcript from this would take around two weeks, imaging and analysis of embryos injected with this mRNA would take another four weeks. Therefore, in total, the suggested experiment would take around 6 weeks. Although the alternative experiment, that is, making a carboxypeptidase domain mutant of vash-1 would be a better choice in terms of reproducibility and long-term use of a stable line, it would admittedly take a relatively larger amount of time. Therefore, the ultimate choice would depend on the authors.

We will investigate this further by cloning and expressing a mutated *vash-1* cDNA which translates a validated catalytically dead Vash-1 (Nieuwenhuis et al., 2017). However, this mutant has not been shown to function as dominant negative, so it is unclear whether it can be used as a dominant negative mutant.

3 . Both the data and methods are presented in a way that ensures reproducibility. The statistical analysis is very well done, in that the authors were very prudent in their choice of statistical tests. However, in many figures and subfigures (Fig. 2B, H-J; Fig. 3G, J, K, N; Fig. 4J; Fig. 5J), the number of replicates was not mentioned and instead only the sample size was stated. Whether this was just an oversight or if it should be taken to mean that the analysis was performed on just one replicate is unclear. The authors need to clarify this aspect of their analysis. Further, In Fig. 2H-J, Fig. 3G,J, K, N and Fig. 4J, the total number of data points in control MO vs vash-1 KD seem to be quite different. In other words, there seem to be a lot more data points in one experimental condition than the other. Does this difference fall within the acceptable range? If the authors were to compare a similar number of data points between the two experimental conditions, would the results of the statistical analysis still be the same?

We apreciate this comment and clarified the replicate numbers in the figure legends: Fig. 2B- 3 replicates (page 25), Fig. 2 H-J- quantification is 1 replicate (page 26), Fig. 2 D-G is representative of 3 replicates (page 25). Fig. 3 G,J,K,N - quantification is from 1 replicate (page 26), Fig. 3 B,C,E,F,H,I are representative of 2 experimental replicates (page 26). Fig. 4J - quantification is 1 replicate (page 27), Fig. 4 A-F is representative of 3 replicates (page 27). Fig. 5 J correspondes to 1 replicate (page 28).

We plan to increase replicates and numbers in quantifications shown in Fig. 3 G,J,K,N and Fig. 5 J as they are relevant for the conclusions of the manuscript, and adapt the text.

The quantifications of immunostaining signals are comparable between different samples of the same experiment but technically not easy accross different experiments, due to some variability of the immunostaining. However, the pattern we report in the quantifications and representative pictures is consistentely detected (reduced dTyr signal upon *vash-1* KD in Fig 2 D-G; higher dTyr intensity in secondary rather than primary sprouts in Fig. 4 A-F). We added in the legend that the pictures of the embryos in these figures are representative of 3 biological replicates (see page 25 and 27).

We recognise the unequal sample size in control and *vash-1* KD groups in Fig. 2H-J, Fig. 3G,J, K, N and Fig. 4J. Generally, the *vash-1* KD group shows more variance than the control group (see Fig. 3 J-N, 4J for example), hence the reason why we analysed a higher sample size. In the planned experiments (repeating quantifications of Fig. 3 J-N), we will analyse a similar number of embryos.

© 2021. Published by The Company of Biologists under the terms of the Creative Commons Attribution License (https://creativecommons.org/licenses/by/4.0/).

We corrected the figure legend of 2 H-J on the number of ISVs - 108 ISVs from 7 embryos for control and 150 ISVs for *vash-1* KD, from 9 embryos (see page 26).

4 . The authors only provide KD data on the function of vash-1 using morpholinos. According to several recent guidelines concerning the use of morpholinos, this is not widely accepted in the zebrafish community as sufficient to provide robust insight into gene function. Please refer for example to the following publication: Guidelines for morpholino use in zebrafish, Stainier et al., PLOS Genetics, 2017. The generation of a vash-1 mutant is a necessary requirement for backing up morpholino KD data. Further, even though the authors state that embryos were selected on the pre-established criteria that they have normal morphology, beating heart, and flowing blood, certain morphological differences between control MO injected and vash-1 KD embryos could be observed in some figures. In Fig. 2D, F and Fig. 5A, B, E, F the vash-1 KD embryos seem smaller (extend of the dorso-ventral axis) than control MO injected embryos. The authors need to provide images showing the overall morphology of morpholino injected embryos and need to provide evidence that morpholino injections do not cause developmental delays.

We agree that a mutant would be desirable to validate the phenotypic analysis of the morpholinos used, and would also allow for further analysis. However, this is not achievable within a reasonnable time frame, especially in the context of current work restrictions. We have added a sentence about the need to confirm the loss of function phenotype with *vash-1* mutants in the discussion (see page 14).
In addtion to the two morpholinos currently used to knockdown *vash-1* expression, we will use an ATG morpholino to further investigate our observations and hypothesis regarding the role of *vash-1* in lymphatic vessels formation. We will also validate it by westernblot and attempt to rescue it with mRNA.

We added a supplementary figure with pictures and quantifications of antero-posterior (Sup. Figure 1 C) and dorso-ventral length (Sup. Figure 1 D) of the analysed control and *vash-1* morpholino injected embryos' development at 24, 34, 52 and 4dpf which shows no significant developmental delay and morphological defect. There is some occurrence of curvature of the tail at 34-52 hpf.
We added a sentence in the Methods section (pages 10) to clarify the morphant's morphology and dosage- response curves.

We observe a 1-2 hour developmental delay of both the control and the *vash-1* KD embryos compared to uninjected wild-type embryos, which led us to chose the 52 hpf time point to investigate the PLs. In uninjected embryos they are usually developed by 48hpf (Hogan et al., 2009).

Fig. 2 D shows a more anterior region of the zebrafish trunk than Fig. 2F (the tail has a smaller dorso-ventral length)- we will provide more comparable pictures from the same region.
Fig. 5B is slightly tilted - we will provide a picture with the same orientation.
Fig. 5 E and F have a similar length from dorsal aorta to the dorsal longitudinal anastomotic vessel. However, we appreciate a difference in the sub intestinal vascular plexus (SIVP), which is consistently underdeveloped in the *vash-1* KD embryos.

© 2021. Published by The Company of Biologists under the terms of the Creative Commons Attribution License (https://creativecommons.org/licenses/by/4.0/).

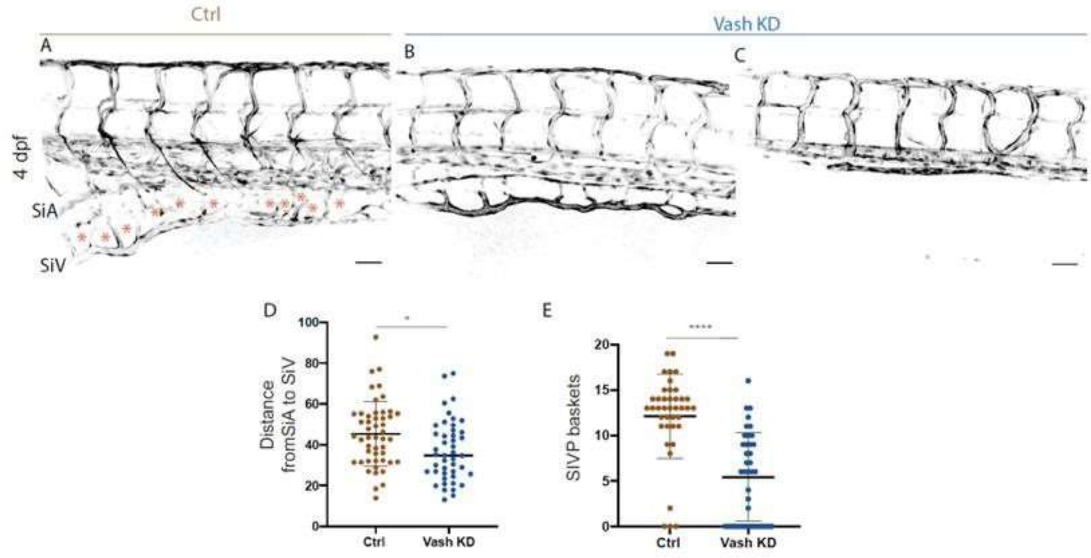

Figure 2- *vash-1* deficient embryos show underdeveloped intestinal vascular system at 4 dpf.

Minor comments:

a. The authors should back their qPCR data for *vash-1* expression (Figure 1) by standard mRNA in situ hybridization, given the large degree of variability in *vash-1* expression. Do they observe a dynamic expression in the vasculature using this technique?

We agree with the reviewer that an in situ hybridization would be beneficial to understand the expression pattern of *vash-1* in wild type embryos. Accordingly, we will look at *vash-1* expression by *in situ* hybridization in WT embryos.

b. The number of nuclei per sprout in Fig. 3J does not correspond with the number of divisions per sprout presented in Fig. 3K. The authors observe one or two cell divisions per sprout in ctr MO injected embryos (Fig. 3K), however, Fig. 3J shows that the majority of ctr. sprouts contains only one cell. This is even more dramatic for *vash-1* MO injected embryos, which can have up to four divisions, therefore should contain six cells. However, the maximum number of cells the authors report is three to four cells. How do these observations go together?

We believe these quantifications are not contradicting. The number of endothelial nuclei was assessed just prior to the connection to the ISV and the cell division quantification was done in a time-lapse from the time of secondary sprout emergence until the resolution of the 3-way connection. It is expected that there are more cell divisions during a longer time frame, as cells migrate dorsally or ventrally out of the sprout.

c. Fig. 5I and J have the same data points for control MO and *vash-1* MO1. Does this mean that both graphs are from the same experiment? If so, the authors could combine the two graphs into one. If the two graphs are not from the same experiment, both would need to have independent controls.

Fig 5 I and J are indeed from the same experiment. They are now combined into one graph (see Fig. 5 J).

d. The percentage of somites with PLs in *vash-1* MO1 injected embryos in Fig. 5I is half the value shown in Fig. 5C. Although this kind of variability might be expected in biological samples, perhaps the authors could briefly discuss the issue and its implications on reproducibility in the manuscript so as to have the readers be aware of it, especially since the rescue of the *vash-1* morpholino phenotype back to 50% from 25% is the same value the authors observed in the *vash-1* KD alone in Fig. 5C. Here the value is 50% for the morpholino injection.

© 2021. Published by The Company of Biologists under the terms of the Creative Commons Attribution License (https://creativecommons.org/licenses/by/4.0/).

We added a sentence discussing the phenotypic variability in the discussion (see page 16), and we added a dosage response curve for the PLs (Sup. Figure 1 F), showing that embryos injected with the same amount of morpholino show variability in the percentage of somites with PLs at 52hpf. We added a more representative picture of PLs for *vash-1* morphant in Fig. 5I (<50% somites have PLs).

e. The Y-axis label is missing in Fig. 2H and Fig. 4J. Figure 5D lacks bars showing median and standard deviation.

Y-axis of Fig. 2H and 4J correspond to ratios, which have no units. Nontheless, we added AU/AU to these graphs to make it clearer. We added the bars in Fig. 5D.

f. It would help to have an inference or conclusion at the end of each results section.

We added one conclusion sentence per results section (see pages 11-14).

Reviewer #3 (Significance (Required)):

Conceptual: As per my knowledge, this is the first study that looks at microtubule modifications in the context of a vertebrate organism past the gastrulation stage, as opposed to similar studies that have been done in cell culture or invertebrates (S. cerevisiae, C. elegans and D. melanogaster). Moreover, this study is one of few that address a novel link between the cytoskeleton and the process of cell fate specification.

Previous studies have separately shown that Vash-1 limits angiogenesis and detyrosinates MTs. The current study combines the two observations in the context of endothelial cells, and hypothesizes that perhaps the function of Vash-1 in limiting angiogenesis and at the same time promoting lymphatic development could be due to its role in MT modification at the molecular level and the consequent effect of this on cell division and/or fate specification at the cellular level. In short, this study aims to connect the long-standing gap in knowledge between cytoskeletal modifications and cell dynamics (in particular, division and specification) in a vertebrate organism. I therefore believe that the current study would be an exciting finding for research communities that study cytoskeletal influence on cellular dynamics and also those in the broad area of vascular biology.

My field of expertise relates to vascular biology, specifically developmental angiogenesis and the behavior of endothelial cells in zebrafish.

References

Barisic, M., Silva E Sousa, R., Tripathy, S. K., Magiera, M. M., Zaytsev, A. V., Pereira, A. L., Janke, C., Grishchuk, E. L., & Maiato, H. (2015). Microtubule detyrosination guides chromosomes during mitosis. *Science*, *348*(6236), 799-803. https://doi.org/10.1126/science.aaa5175

Hogan, B. M., Bos, F. L., Bussmann, J., Witte, M., Chi, N. C., Duckers, H. J., & Schulte-Merker, S. (2009). Ccbe1 is required for embryonic lymphangiogenesis and venous sprouting. *Nature Genetics*, *41*(4), 396-398. https://doi.org/10.1038/ng.321

Isogai, S, Horiguchi, M., & Weinstein, B. M. (2001). The vascular anatomy of the developing zebrafish: an atlas of embryonic and early larval development. *Developmental Biology*, *230*(2), 278-301. https://doi.org/10.1006/dbio.2000.9995

Isogai, Sumio, Lawson, N. D., Torrealday, S., Horiguchi, M., & Weinstein, B. M. (2003). Angiogenic network formation in the developing vertebrate trunk. *Development*, *130*(21), 5281-5290. https://doi.org/10.1242/dev.00733

Kimura, H., Miyashita, H., Suzuki, Y., Kobayashi, M., Watanabe, K., Sonoda, H., Ohta, H.,

© 2021. Published by The Company of Biologists under the terms of the Creative Commons Attribution License (https://creativecommons.org/licenses/by/4.0/).

Fujiwara, T., Shimosegawa, T., & Sato, Y. (2009). Distinctive localization and opposed roles of vasohibin-1 and vasohibin-2 in the regulation of angiogenesis. *Blood*, *113*(19), 4810-4818. https://doi.org/10.1182/blood-2008-07-170316

Koltowska, K., Lagendijk, A. K., Pichol-Thievend, C., Fischer, J. C., Francois, M., Ober, E. A., Yap, A. S., & Hogan, B. M. (2015). Vegfc Regulates Bipotential Precursor Division and Prox1 Expression to Promote Lymphatic Identity in Zebrafish. *Cell Reports*, *13*(9), 1828-1841. https://doi.org/10.1016/j.celrep.2015.10.055

Liao, S., Rajendraprasad, G., Wang, N., Eibes, S., Gao, J., Yu, H., Wu, G., Tu, X., Huang, H., Barisic, M., & Xu, C. (2019). Molecular basis of vasohibins-mediated detyrosination and its impact on spindle function and mitosis. *Cell Research*, *June*. https://doi.org/10.1038/s41422-019-0187-y

Lim, A. H., Suli, A., Yaniv, K., Weinstein, B., Li, D. Y., & Chien, C. Bin. (2011). Motoneurons are essential for vascular pathfinding. *Development*, *138*(21), 4813. https://doi.org/10.1242/dev.075044

Nicenboim, J., Malkinson, G., Lupo, T., Asaf, L., Sela, Y., Mayseless, O., Gibbs-Bar, L., Senderovich, N., Hashimshony, T., Shin, M., Jerafi-Vider, A., Avraham-Davidi, I., Krupalnik, V., Hofi, R., Almog, G., Astin, J. W., Golani, O., Ben-Dor, S., Crosier, P. S., … Yaniv, K. (2015). Lymphatic vessels arise from specialized angioblasts within a venous niche. *Nature*, *522*(7554), 56-61. https://doi.org/10.1038/nature14425

Nieuwenhuis, J., Adamopoulos, A., Bleijerveld, O. B., Mazouzi, A., Stickel, E., Celie, P., Altelaar, M., Knipscheer, P., Perrakis, A., Blomen, V. A., & Brummelkamp, T. R. (2017). Vasohibins encode tubulin detyrosinating activity. *Science*, *358*(6369), 1453-1456. https://doi.org/10.1126/science.aao5676

Submission to Development

First decision letter

MS ID#: DEVELOP/2020/194993

MS TITLE: Vasohibin-1 selectively regulates secondary sprouting and lymphangiogenesis in the zebrafish trunk

AUTHORS: Marta Bastos de Oliveira, Katja Meier, Baptiste Coxam, Ilse Geudens, Simone Jung, Anna Szymborska-Mell, and Holger Gerhardt

Thank you for transferring your paper to Development from Review Commons.

I have now read the referees' reports on the above manuscript and your response to them and have reached a decision. The referees' comments are appended below, or you can access them online: please go to BenchPress and click on the 'Manuscripts with Decisions' queue in the Author Area.

My main issue of concern is that you describe new phenotypes based upon the use of morpholinos. Given well recognised problems with MOs causing non-specific phenotypes, the journal is very cautious about publishing zebrafish papers using these reagents without very rigorous controls (which can be more challenging than generating mutants). I would normally flag this issue at the point of submission but of course, in this case, we only now see your manuscript subsequent to reviews and your response. I agree with your comment that it would take a long time to make a new mutant, especially at a time when labs and animal facilities are not fully operational. Given this situation, I suggest that in addition to performing more robust morpholino controls, you also analyse phenotypes in embryos in which you have used Crispr/cas9 to target the gene. This F0

© 2021. Published by The Company of Biologists under the terms of the Creative Commons Attribution License (https://creativecommons.org/licenses/by/4.0/).

crispr approach can be very effective and there are a few recent papers on the approach including one on BioRxiv from Jason Rihel's group. Adding this approach has the advantage that it is a very different method to morpholinos and so the likelihood of obtaining equivalent off-target phenotypes is reduced. You can also assess the effectiveness of the guides to remove wild-type alleles. You can, of course, also use the same reagents, albeit likely at lower concentrations, to generate stable lines. Other than this issue, I think that your suggested responses to reviews are reasonable. Given that you will be performing some new experiments, your revised paper will be re-reviewed by one or more of the original referees.

We are aware that you may have limited access the lab to undertake experimental revisions. If it would be helpful, we encourage you to contact us to discuss your revision in greater detail. We will then provide further guidance. Please also note that we are happy to extend revision timeframes as necessary.

Please attend to all of the reviewers' comments and ensure that you clearly highlight all changes made in the revised manuscript. Please avoid using 'Tracked changes' in Word files as these are lost in PDF conversion. I should be grateful if you would also provide a point-by-point response detailing how you have dealt with the points raised by the reviewers in the 'Response to Reviewers' box. If you do not agree with any of their criticisms or suggestions please explain clearly why this is so.

First revision

Author response to reviewers' comments

Reviewer #1 (Evidence, reproducibility and clarity (Required)):

The manuscript entitled "Vasohibin-1 mediated tubulin detyrosination selectively regulates secondary sprouting and lymphangiogenesis in the zebrafish trunk" by de Oliveira investigates the function of the carboxylpeptidase Vasohibin during the formation of the zebrafish trunk vasculature and reports a requirement of Vasohibin for secondary sprout formation and in particular the formation the lymphatic vasculature.

Having established the expression of Vasohibin in sorted ECs of 24 hpf embryos, the remaining study addresses the function of Vasohibin in this cell type. It is largely based on the use of a splice-site interfering morpholino. Particular commendable is the analysis, demonstrating that the KD of *vash1* indeed results in a significant reduction of detyrosination in endothelial tubulin. Findings in the vascular system then include:
(i) the detection of increased division and hence supernumerous cells occurring selectively in 2nd sprouts from the PCV; (ii) an increased persistence of the initially formed 3 way connections with ISV and artery; (iii) reduced formation of parachordal lymphangioblasts and (iv) a reduced number of somites with a thoracic duct segment; (v) frequent formation of lumenized connections between PLs (where present) and ISV. To demonstrate specificity, the approach was repeated with a different morpholino and defects were partially rescued by MO-insensitive RNA.

Possible additional and relevant information could include data on a *vash1* promotor mutant to independently verify the MO-based functional analysis. Mutants would also allow analysis of further development, are the defects leading to the demise of the fish or is a later regeneration and normalization of the lymphatic vasculature observed?

We agree that a mutant would be desirable to validate the phenotypic analysis of the morpholinos used, and would also allow for further analysis.
We have therefore used CRISPR/Cas to generate F0 embryos, and investigated the embryonic phenotype of the mutants. At 2dpf, the mutants lack PLs (Figure 6 J) and exhibited also some other features we observe in the morphants such as higher diameter veins. We added the genotyping of these mutants in sup. fig. 4. We are currently growing these mutants to perform

© 2021. Published by The Company of Biologists under the terms of the Creative Commons Attribution License (https://creativecommons.org/licenses/by/4.0/).

further analysis on longer term defects, as the suggested by the reviewers.

In addition, are other lymphatic vessel beds like the cranial lymphatics affected?

Using the *Tg[fli1a:EGFP]^{y7}* line, we have not been able to identify apparent differences in other vascular beds including the cranial lymphatics.

PLs have been demonstrated to be at least partially guided in their movement by the CXCR4/SDF1 system and SVEP1. Has the expression of these factors been tested in *vash1* KDs?

We appreciate this question by the reviewer since the failure of PL development is the most consistent phenotype we observe in the *vash1* morphants. We investigated the expression of *cxcr4a* by *in situ* hibridization upon *vash1* knock-down. The expression pattern and level was similar in control and *vash1* morphants (Figure 1 A,B), so we believe this system is not implicated in the *vash1* morphants.

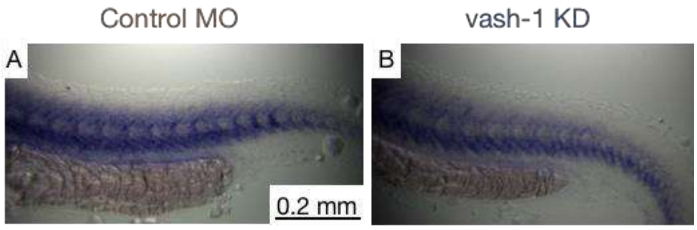

Figure 1- *cxcr4* expression in control and *vash1* injected MOs. **N=20/each**

With regards to the frequently observed connections of PLs and ISVs in *vash1* morphants, can the proposed lumen formation of these shunts be demonstrated e.g. by injection of Q-dots or microbeads into the circulation?

We quantified occurences of ISV-ISV connections in Tg(Fli1a:EGFP,kdr-l:ras-Cherry) (Supplementary figure 3A) and performed qDots injections in 4 dpf embryos (Supplementary figure 3 B-D), to investigate whether these ISV-to-ISV connections are lumenised. We found that the vash1 morphants exhibit three times more ISV-ISV connections than the controls, and moreover, that two thirds of the morphants' ISV-to-ISV connections exchibit contain qDots, and are therefore lumenised (Supplementary figure 3 B-D, arrow heads show lumenized ISV-ISV connections).

Concerning the mechanisms of these defects, is it possible to analyse the asymmetric cell division leading to 2nd sprouts in greater detail? Is the same number or are more cells sprouting form PCV and can the fli1ep:EGFP-DCX cell line in fixed samples be used to identify the spindle orientation in dividing cells?

We agree with the reviewer that investigating the cell division at this stage would enrich the manuscript. We used the *Tg[fli1ep:EGFP-DCX]* fish line to investigate the spindle in control and *vash1* KD embryos. This analysis proved extremely difficult as we need high enough resolution to adequately pick up the spindle from prometaphase to anaphase, and need to catch the right moment with high enough temporal resolution. A preliminary analysis showed that we needed to perform the time-lapse acquisition in intervals of 2-2,5 minues to catch a displacing spindle. However, the small field of view and high temporal resolution meant that we can only gamble on whether or not a time-lapse movie of a given field of view will manage to contain the cell division event in secondary sprouting. After long and tedious repeats, we managed to catch 10 number of events in control and 5 number of events in vash1 MO embryos (Figure 2 of this letter).

We analysed the displacement of the spindle from its position in prometaphase until anaphase and the angle of spindle plane relative to the axial orientation of the PCV. We observed a greater displacement of the spindle in controls, potentially indicating a asymmetric cell division in the

© 2021. Published by The Company of Biologists under the terms of the Creative Commons Attribution License (https://creativecommons.org/licenses/by/4.0/).

controls, absent in *vash1* morphants (Figure 2 of this letter). We also observed mostly cell divisions with the spindle oriented perpendicularly to the PCV axis in the control embryos, and an increased incidence of parallel cell divisions (defined by angles lower than 45 or higher than 135) in *vash1* morphants (Figure 3 of this letter). However, given the small number of events we managed to acquire we feel this is not sufficient to draw firm conclusions. This will likely need additional method development with new transgenic lines to fully address the question of assymetric cell division.

We have removed unpublished data provided for the referees in confidence.

Figure 2- Spindle displacement from prometaphase to before anaphase.

We have removed unpublished data provided for the referees in confidence.

Figure 3- Spindle orientation, before anaphase.

Regarding the question of the number of sprouts versus cells within sprouts, we analysed the time-lapse acquisitions from Figure 3 and 4 carefully, to identify the stem of each secondary sprout in the *vash1* KD embryos. We found that there is not an increase of independentely activated cells sprouting (Fig. 3 H), but rather an increase of frequency of persistent protrusions splitting the secondary sprout reaching for neighbooring ISVs (quantified in Fig. 3 G) and more cells sprouting through the same number of secondary sprouts (quantified in Fig.4 C).

Minor issues:

Page 5, Mat & Meth, please spell out PTU at its first mention.

This has been corrected accordingly (see page 5).

Page 6 Mat & Meth, Secondary sprout and 3-way connection parameters: The number of nuclei was assessed in each secondary sprouts (del s, singular) just prior...

This has been corrected accordingly (see page 6-7).

Page 16, 8th line from bottom: Recent work demonstrated that a secondary sprout either contributes (add s) to remodelling a pre-existing ISV into a vein, or forms (add s)a PLs (Geudens et al., 2019).

This has been corrected accordingly (see page 20).

Page 25, Legend to Fig. 2D-G: "...G,G' shows quantification of dTyr signal upon *vash1* KD..." Fig2 G,G' show immunostaining rather than quantification of the dTyr signal, which is shown Fig. 2H-J

This has been corrected accordingly (see page 30).

Fig. 1D / Fig. 2H-J please increase weight of the error intervals and / or change colour for improved visibility

This has been corrected accordingly (Fig. 1D and 2H-J), and we added n.s. to Fig. 1D.

Reviewer #1 (Significance (Required)):

Taken together the manuscript is comprehensively written and the study provides a conclusive analysis of the MO-mediated KD of Vasohibin in zebrafish embryonic development presenting significant novel findings.

Known was a generally inhibitory function of Vasohibin on vessel formation and its enzymatic activity as a carboxylpeptidase responsible for tubulin detyrosination, affecting spindle function and mitosis. New is the detailed analysis of the Vasohibin KD on zebrafish trunk vessel

© 2021. Published by The Company of Biologists under the terms of the Creative Commons Attribution License (https://creativecommons.org/licenses/by/4.0/).

formation and the description of a selective impairment of 2nd sprout formation.

The manuscript is of interest for vascular biologists.

REFEREES CROSS-COMMENTING

I fully concur with the comments of reviewer #2, all three reviews find that this study is of significant interest to the vascular biology community as the relevance of tubulin detyrosination for developmental angiogenesis has not been investigated. Also all three reviews highlight the potential limitations of the use of splice morpholinos (suggested alternatives include ATG morpholinos and CRIPR mutants), the requirement to provide further evidence for a endothelial cell autonomous defect and the need to clarify some of the data representation.

Reviewer #2 (Evidence, reproducibility and clarity (Required)):

Summary:

The manuscript by Bastos de Oliveira et al. describes an important investigation of the endothelial tubulin detyrosination during vascular development. Namely, they found detyronised microtubules in secondary sprouts, which is absent in MO-vash1 treated embryos. The authors use the vash1 morpholino approach to uncover the developmental consequences of suppressed detyrosination in angiogenesis and lymphangiogenesis in vivo in zebrafish. By a combination of transgenic lines, immunohistochemistry and time-lapse imaging, Bastos de Oliveira et al., have found that Vash1 is a negative regulator of secondary sprouting in zebrafish. The authors showed that in the absence of Vash1 more cells are present in the secondary sprouts due to increased cell proliferation; however lymphatic vascular network fails to form. The current manuscript requires additional experimental evidence to support the conclusions. Please see below the major technical concerns and minor comments.

Major comments:

-This study is based on analysis of the phenotypes observed in embryos injected with vash1 morpholino. The authors use two different types of splice morpholinos, perform rescue experiments with RNA, and validate one MO-vash1 with western blot. Morpholinos are not trivial to work with, and the results are variable hence additional controls need to be included, as following the recommendation put together by the zebrafish community (Stainier et, al., Plos Genetics, 2017). As the severity of the phenotypes comparing MO1 with MO2 is different and MO-vash1 embryos appear developmentally delayed (Figure 2D-F and 5E-F overall size seem to be affected), additional MO is required, for example, ATG-MO or generation of CRISPR mutant would be favourable. All the morpholino used need to be validated using an antibody, RT-PCR and qPCR. It is essential to carry out the rescue experiments for all the MO used in this study and following the guidelines. Including the dose-response curve, data would be informative.

We agree with the reviewer and the recommendations of the zebrafish community. We investigated the phenotype with additional morpholinos, such as the ATG-Morpholino suggested by the reviewer. We could confirm the absence of PLs in all morphants tested (see Supp. Figure 2 D-E). We also supply more validation for the morpholinos, including RNA rescue (Figure 6C, supp. Figure 2 B-C) and westernblots for all morpholinos (Figure 2 B and Supplementary Figure 2 B-C).
We added dose-response curves (Supp. Figure 1 E,G-I) and a developmental morphology assessment for the morpholino 1 (Supp. Figure 1 A,B).
Given our extensive analysis of the effects of *vash1* KD, we believe the embryos in 2F are not developmentally delayed. However, the image in figure 2F did give that impression because it was from a more posterior part of the trunk, due to an oversight. We added pictures of the same trunk region (Fig.2D- F). We nonetheless supply a supplementary figure 1 showing and quantifying the development of the analysed *vash1* morphants.
We also agree that mutants are favourable to validate the phenotypes observed by the

© 2021. Published by The Company of Biologists under the terms of the Creative Commons Attribution License (https://creativecommons.org/licenses/by/4.0/).

morpholinos. Therefore used CRISPR/Cas to generate F0 embryos, and investigated the embryonic phenotype of the mutants. At 2dpf, the mutants lack PLs (Figure 6 J) and exhibited also some other features we observe in the morphants such as higher diameter veins. We added the genotyping of these mutants in sup. fig. 4.

-In addition to EC, the levels of dTyr are lower in MO-vash1 in neural tube and neurons spanning the trunk (Figrue 2 D-G'). These have been previously shown to be important for secondary sprouting. Is it possible that the observed phenotypes in the secondary sprouting are due to defects in these neurons?

We agree with the reviewer that motorneurons are relevant for secondary sprouting pathfinding (Lim et al., 2011) and this should be clarified in the vash1 morphants. In the mentioned publication, the neurons morphology was tightly connected with their ability to guide secondary sprouts. We therefore assessed the morphology of the neurons and their dendrites relevant for pathfinding in *vash1* KD embryos, using the pan-neuronal zebrafish line Tg(3xmnx1:GAL4-VP16)^{ku20tg} ;Tg(14xUAS:mRFP,Xla.Cryg:GFP)^{tpl2}.
Although their detyrosination level is dereased in the vash1 morphants (fig. 2D-G) as pointed out by the reviewer, we did not find any recogniseable morphological differences between the motoneurons of control and vash1 KD embryos (Figure 4 of this letter).

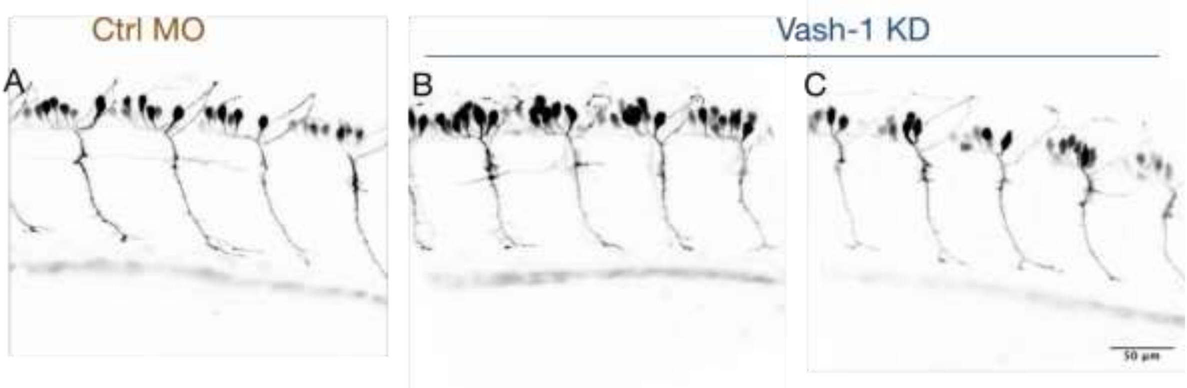

Figure 4 - Motorneurons are unaffected in *vash1* morphants. Tg(3xmnx1:GAL4-VP16)^{ku20};Tg(14xUAS:mRFP,Xla.Cryg:GFP)^{tpl2}embryos injected with Control (A) or vash1 morpholino (B,C) exhibit similar motorneurons. Motorneurons of morphants exhibit some variability, depicted in B,C. Pictures are representative of 2 experimental replicates. N=28 for vash1 KD and N=22 for Control group.

-Embryo number used in this study appears to be low especially in figure 3G, 5D, 5G, to conclude draw conclusions from these experiments, the number of embryos used should be higher than 20. Figure 4J please specify how many embryos were used.

We agree with the reviewer about the low number of experiments quantified in 3G in the first submitted version of this manuscript. We increased the number of embryos per condition to a minimum of 20 embryos and updated the averages in the text for 3G.

In graphs 5D and 5G (now 6D and 6G) each data point is an embryo and more than 20 embryos per condition were used (in 6D 23-35 embryos per condition, in 6G 60-63 embryos/condition), we corrected the legend 6D and 6G and added N numbers in the graphs for clarification.

In 4J (now 5J), 18 embryos were used for primary sprouts (2-3 sprouts/embryo- 52 sprouts quantified) and 12 embryos for secondary sprouts (about 2 sprouts/embryo - 24 sprouts quantified). We corrected the number of control sprouts in the legend and added the number of embryosin the graph to increase clarity.

-The authors hypothesise that VASH acts in the sprouting endothelial cells, based on the Q-

© 2021. Published by The Company of Biologists under the terms of the Creative Commons Attribution License (https://creativecommons.org/licenses/by/4.0/).

PCR in Figure 1. However, in this experiment all EC have been sorted thus this remains ambiguous in which cell types vash1 is expressed. Please provide the expression pattern for vash1 across the developmental stages the phenotypes are observed.

We agree with the reviewer that it would be beneficial to understand the expression pattern of *vash1* in wild type embryos. We performed an *in situ* hybridization for *vash1* mRNA that revealed expression at 24 and 34hpf in the dorsal aorta and perivascular tissue (Figure 1 E-G).

-Throughout the manuscript the authors refer the lymphatic identity, however, there is no evidence in the paper that the identity status has been assessed. To support these claims Prox1 immunohistochemistry or analysis of prox1 expression in the reporter line would be appropriate.

We agree with the reviewer and teamed up with Koltowska lab for the Prox1 immunostaining (Koltowska et al., 2015) in *vash1* KD embryos at 34 hpf to investigate Prox1 levels upon *vash1* KD. We found evidence for an increased frequency of neighbouring cells expressing Prox1, including ECs in the PCV which are normally Prox1 negative (added in Fig. 4 E-G). This would fit with a scenario in which cells divide in the PCV and instead of aquiring a high and low Prox1 levels during lymphovenous specification in the PCV, acquire similar levels and behaviour. This result is consistent with the increased number of cells in the sprout in *vash1* morphants (Fig. 4 A-C).

Minor comments:

-The authors refer to the literature where overexpression of VASH suppresses the angiogenesis. As the RNA injections were used in rescue experiments, the data of vash1 RNA injections into the wild-type embryos would be beneficial.

We appreciate this question from the reviewer and have therefore peformed three replicates of injections of *vash1* RNA into a control morpholino injected embryos (Figure 5 of this letter) and did not observe a significant loss of PLs at 52 hpf.

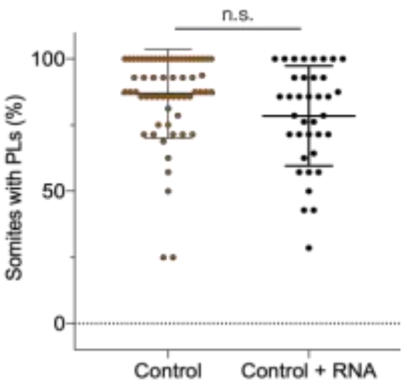

Figure 5 - *vash1* overexpression did not lead to a loss of PLs at 52 hpf. *Tg[fli1a:EGFP]*^{y1}embryos injected with control and vash1 morpholino did not show any difference in PL number. N= 60 control embryos and N= 40 *vash1* KD embryos

-In figures 2J, 3J, 3K, 3N, 4J, 5C, 5D and 5G the N number was set for examples as the number of sprouts, the number of somites with TD, number of ISV. To strengthen the observation in the manuscript quantification of the sprouts, PL, vISVs and lymphatic phenotypes with N set as the number of embryos would be more informative. Indicating the number of embryos used, in the graphs, would be helpful.

We agree with the reviewer that the embryo numbers culd be more clearly stated and have therefore added embryo numbers in all legends and graphs.

© 2021. Published by The Company of Biologists under the terms of the Creative Commons Attribution License (https://creativecommons.org/licenses/by/4.0/).

In graphs 3J, 3K (now 4C), 4J (now 5 J) each data is a sprout, a cell division or an ISV, so we included these numbers in the graphs too. In all other mentioned graphs, each data point refers to an embryo (percentage of somites with PLs/embryo, percentage of the existing PL/embryo connected to a venous ISV and percentage of somites with a TD segment/embryo).

-In Figure 5A, B and D the authors quantify what they refer to as a lumenised connection between the vISVs and PL. In the control image (second star), a somewhat lumenised structure is present, clarification of how the scores were set is missing.

In Fig. 5C (now 6C) we show a quantification of the percentage of somites with PLs per embryo, by counting the PLs identified with an asterisk in Fig. 6 A-B.
To clarify the quantification of Fig. 5D (now 6D): we quantified the proportion of PLs associated/connected with venous ISVs (see Methods section page 6), by 52 hpf in control and *vash1* morphants.
We discuss lumenized connections between ISVs at 4 dpf, present in Figure 6F,F‘. We added a quantification of ISV-ISV connections of control / vash1 KD embryos in Sup. Fig. 3 A. We demonstrated their lumenisation by detecting quantum dots (Sup. Fig. 3 B-D).

-In Figure 3 E and F the authors show the excessive sprouting phenotype between controls and Mo-vash1. The images presented are taking from different parts of the embryos (middle of the trunk vs plexus region), hampering the comparison between the two groups. The quantification of the phenotypes in both experimental groups should be in the same region of the embryo, as the local difference can occur. It is key to provide representative images to support these observations.

The images presented are representative of the phenotype quantified, and the time-lapses were done in comparable regions of the zebrafish trunk (+- 1-2 somites in both groups due occurring drift during image aquisition), making the comparison possible. Specifically, the identified secondary sprouts in these pictures are from the same area of the trunk.

-Figure 1D the vash1 expression levels in EC seem very variable in this graph, therefore no conclusion can be drawn from this data, especially as the authors do not provide the p-values.

We added n.s. in the graph, to make it clear that the difference between developmental stages is not significant. We believe that the non significance is potentially due to high biological variability between embryos, detected with the two sets of primers. We believe that presenting this biological variability is of importance to the readers. The additional in situ hybridization results point at constant *vash1* expression in the brain throughout the stages tested (24, 34, 48 hpf- not shown) and endothelial expression only at 24 and 34 hpf (Fig. 1 F-H), consistent with the result from the qPCR.
We wrote on page 12 about this result: „During the sprouting phase (24hpf), *vash1* expression was 5-7 times higher in endothelial than in non-ECs, decreasing at 48 hpf (Fig. 1C-D). Although these results are not significant, they were independently confirmed with a second primer set.”. The only conclusion we made from this data is that *vash1* is expressed in the zebrafish endothelium during development, as we now added in the results (page 18).

-In the introduction, the authors state: 'Although primary and secondary sprouts appear morphologically similar, with tip and stalk cells' - Please provide the reference that supports the claim that secondary sprouts have tip-stalk cells morphology/organisation.

We appreciate the question of the reviewer. Although some studies identify shared and distinct molecular regulation of primary and secondary sprouts, and revealed morphological similarity, there is no formal proof that secondary sprouts have a tip-stalk cell morphology. Given that this is not relevant for the central findings of the work, we modified the sentence and added a reference “Although primary and secondary sprouts appear morphologically similar, ~~with tip and stalk cells~~ with acto-myosin protrusions (Sumio Isogai et al., 2003)…” See page 3.
We also updated the discussion for consistency: “Although the cellular mechanisms of primary and secondary sprouting in zebrafish appear very similar, ~~with tip cell selection and~~

© 2021. Published by The Company of Biologists under the terms of the Creative Commons Attribution License (https://creativecommons.org/licenses/by/4.0/).

~~guided migration and stalk cell proliferation,~~ secondary sprouting utilises alternative signalling pathways and entails a unique specification step that establishes both venous ISVs and lymphatic structures." (see page 19)

-The authors refer the increased cell division phenotypes observed in the movies, however, the movie files have not been available to the reviewers.

We added movies of cell divisions during secondary sprouting in control and *vash1* morphants (sup. Movies 1 and 2).

Reviewer #2 (Significance (Required)):

This is an important study as uncovering the mechanistic details of angiogenic and lymphangiogenic negative regulators is of high value with the potential for therapeutic developments. To date, Vash1 has been only studied in the context of tumour angiogenesis, vasculature in diabetic nephropathy and pulmonary arterial hypertension, and it remains unclear what is its role during development and how does it regulate vascular network formation. The tyrosination status of microtubule in endothelial cells is understudied. This study revealed, previously uncharacterised detyrosinated microtubules in endothelial cells in vivo. And further dissects how this process might be regulated, brings unique insights into the vascular biology field and beyond. Thus, delving into the cell biological mechanism such as microtubule dynamics and modification in vivo in embryo context is a significant step forward in setting new standards in the field.

I am developmental biologist who has experience in model organisms such as zebrafish and mouse. The main focus of my work is on developmental angiogenesis and lymphangiogenesis.

REFEREES CROSS-COMMENTING

After reading the other reviews comments, it seems that we all agree that this study is of high value to vascular biology field and beyond bringing novel findings.

Importantly the reviewers' comments are in line with each other and have identified several commonalities that should be addressed. Such as:
Further validation of Morpholinos, or using alternative methods to replicate the findings.
additional evidence that the observed phenotypes are primary due to vash1 requirement within EC, and not due to the secondary effect in other cells such as CXCR4/SDF1 system and SVEP1, neurons or general delay of the embryos
Further evidence of for VASH expression pattern the number of embryos used in the experiments, and how the data is represented.

Reviewer #3 (Evidence, reproducibility and clarity (Required)):

Vasohibin-1 (Vash1) is known to detyrosinate microtubules (MTs) and limit angiogenesis. Using in vivo live imaging and whole mount immunofluorescence staining of zebrafish trunk vasculature, Bastos de Oliveira et al. show that the MT detyrosination role of Vash1 is conserved in zebrafish and that Vash1 is essential for limiting venous sprouting and subsequent formation of lymphatics. Their findings suggest a role for MT detyrosination in lympho-venous cell specification.

Major comments:

1 . The authors claim that Vash1 regulates secondary sprouting and lymphangiogenesis by detyrosinating MTs. However, no direct evidence of this link is provided in the manuscript. The authors only separately show that knockdown of vash1 affects MT detyrosination and secondary sprouting and lymphangiogenesis. They have not shown a causative effect. The authors should therefore qualify the above stated claim as speculative. In other words, the authors should mention that their data only suggests that disruption of MT detyrosination is the underlying cause

© 2021. Published by The Company of Biologists under the terms of the Creative Commons Attribution License (https://creativecommons.org/licenses/by/4.0/).

for aberrant secondary sprouting and lymphangiogenesis in vash1 KD embryos.

We agree with the reviewer about the lack of evidence to state that the disruption of microtubule detyrosination leads to aberrant secondary sprouting in the first version of the manuscript.

With this in mind, we have used CRISPR/Cas to mutate codons that translate into aminoacids critical for the carboxypeptidase function of the Vash1. At 2dpf, these mutants exhibited a lack of PLs (Figure 6 J) as well as some other features we observe in the morphants such as higher diameter veins. These mutants exhibited at least a mutation in Cystein in position 175 (genotyping in sup. fig. 4), critical for Vash1 detyrosination function. Therefore, we believe not only that we increased the validation of the morpholinos, as we provided the first and only evidence of causality with these results. However, we want to remain cautious and avoid overstatements in the conclusions of this manuscript. We added a paragraph discussing this in the manuscript (page 19: T"he fact that we observe a similar defect in PL formation in F0 CRISPants carrying mutations that disrupt its catalytic function, however, strongly suggests that microtubule detyrosination is indeed the mechanism required for adequate lymphovenous patterning.
Nevertheless, future work will need to clarify exactly how microtubule detyrosination controls secondary sprouting and lymphatic specification.").
We also adapted the title: Vasohibin-1 ~~mediated tubulin detyrosination~~ selectively regulates secondary sprouting and lymphangiogenesis in the zebrafish trunk.

2 . In order to provide more compelling evidence for a direct relationship between MT tyrosination and lymphangiogenesis, the authors could try mutating the carboxypeptidase domain of vash1 or overexpressing a dominant negative transcript (that contains a mutated carboxypeptidase domain). If this gives the same phenotypes as the vash1 morphants, it would indicate that the carboxypeptidase activity of Vash1 (in detyrosinating MTs) is responsible for limiting secondary sprouting and promoting specification of lymphatics. This suggested experiment is fairly realistic in terms of both time and resources. For example, since the authors already have the human vash1 cDNA cloned, making a dominant negative transcript from this would take around two weeks, imaging and analysis of embryos injected with this mRNA would take another four weeks. Therefore, in total, the suggested experiment would take around 6 weeks. Although the alternative experiment, that is, making a carboxypeptidase domain mutant of vash1 would be a better choice in terms of reproducibility and long-term use of a stable line, it would admittedly take a relatively larger amount of time. Therefore, the ultimate choice would depend on the authors.

We agree with the reviewer that experiments with a dominant negative clone would be probably the best technique to elucidate the causation of the detyrosination with the vascular phenotypes seen during secondary sprouting. We attempted to generate a such a construct by cloning *vash1* , inducing 2 base pair mutagenesis in *vash1* cDNA, which translates to validated catalytically dead Vash1 (C179A) (Nieuwenhuis et al., 2017). However, we did not succeed at expressing this construct in the zebrafish embryos.
The use of the CRISPR/Cas to generate mutations in codons that were previously validated to be critical for vash1 detyrosination function (the mentioned cysteine) introduces some evidence in the manuscript that the carboxypeptidase function of Vash1 is required for PL formation
Given the limited evidence for causaility, we corrected the sentence in the discussion (page 18): "In this study, we identify Vash1~~-mediated microtubule detyrosination~~ as ~~a cellular mechanism~~ as a novel regulator of EC sprouting from the PCV and the subsequent formation of lymphatic vessels in the zebrafish trunk."

3 . Both the data and methods are presented in a way that ensures reproducibility. The statistical analysis is very well done, in that the authors were very prudent in their choice of statistical tests. However, in many figures and subfigures (Fig. 2B, H-J; Fig. 3G, J, K, N; Fig. 4J; Fig. 5J), the number of replicates was not mentioned and instead only the sample size was stated. Whether this was just an oversight or if it should be taken to mean that the analysis was performed on just one replicate is unclear. The authors need to clarify this aspect of their analysis. Further, In Fig. 2H-J, Fig. 3G,J, K, N and Fig. 4J, the total number of data points in

© 2021. Published by The Company of Biologists under the terms of the Creative Commons Attribution License (https://creativecommons.org/licenses/by/4.0/).

control MO vs vash1 KD seem to be quite different. In other words, there seem to be a lot more data points in one experimental condition than the other. Does this difference fall within the acceptable range? If the authors were to compare a similar number of data points between the two experimental conditions, would the results of the statistical analysis still be the same?

We apreciate this comment and increased some replicates (to a minimum of 3) and increased transparency on the sample size in all graphs and legends. We also added in each methods section how many replicates were done in each section and for all mentioned graphs by the reviewer.
We increased replicates and embryo numbers in quantifications shown in Fig. 3 G-N (now Fig. 3 G,H, J, Fig. 4C,D) and Fig. 5 I (now fig. 6 C), and adapted the text. Regarding the asymmetric number of control/vash1 embryos, we repeated the experiments in 3G-J, 4C-D and included more controls to have a similar number of embryos analysed.
The quantifications of immunostaining signals are comparable between different samples of the same experiment but technically not easy accross different experiments, due to some variability of the immunostaining. Therefore, quantifications from Figure 4 J (now 5 J) are taken from one experiment. However, the pattern we report in the quantifications and representative pictures is consistentely detected (higher dTyr intensity in secondary than in primary sprouts in Fig. 5 A-F). We added in the legend that the pictures of the embryos in these figures are representative of 3 biological replicates (see page 32).

4 . The authors only provide KD data on the function of vash1 using morpholinos. According to several recent guidelines concerning the use of morpholinos, this is not widely accepted in the zebrafish community as sufficient to provide robust insight into gene function. Please refer for example to the following publication: Guidelines for morpholino use in zebrafish, Stainier et al., PLOS Genetics, 2017. The generation of a vash1 mutant is a necessary requirement for backing up morpholino KD data. Further, even though the authors state that embryos were selected on the pre-established criteria that they have normal morphology, beating heart, and flowing blood, certain morphological differences between control MO injected and vash1 KD embryos could be observed in some figures. In Fig. 2D, F and Fig. 5A, B, E, F the vash1 KD embryos seem smaller (extend of the dorso-ventral axis) than control MO injected embryos. The authors need to provide images showing the overall morphology of morpholino injected embryos and need to provide evidence that morpholino injections do not cause developmental delays.

We agree that a mutant would be desirable to validate the phenotypic analysis of the morpholinos used, and would also allow for further analysis. We have therefore used CRISPR/Cas to generate F0 embryos, and investigated the phenotype of the mutants. At 2dpf, the mutants exhibit reduced number of PLs (Figure 6 J, sup. fig. 4) and exhibited also some other features we observe in the morphants such as higher diameter veins.
In addition to the main morpholino used (MO1), we validated the PL absence phenotype with two additional morpholinos (sup.fig. 2 A-E): MO2 targeting a different splice region from MO1, and MO3 disrupts translation of vash1. These morpholinos effectively reduced Vash1, as demonstrated by the westernblots (sup. fig. 2 B-C). Morphants of both morpholinos exhibited less proportion of PLs, a phenotype rescued with vash1 mRNA in MO2. With this, we believe we validated the phenotype of morphants injected with MO1 and increased the confidence in our results.
We overlooked a few figures that contributed to the reviewer perception that the embryos were of different size:
Fig. 2 D showed a more anterior region of the zebrafish trunk than Fig. 2F (the tail has a smaller dorso- ventral length)- we provide new comparable pictures from the same region.
Fig. 5B (now Fig. 6B) was slightly tilted - we now provide a picture with the same orientation.
Fig. 5 E and F (now fig. 6 E and F) have a similar length from dorsal aorta to the dorsal longitudinal anastomotic vessel. However, we appreciate a significant difference in the sub intestinal vascular plexus (SIVP), which is consistently underdeveloped in the *vash1* KD embryos. We added this result in the sup. fig. 5.
To make clear the size of the morphants, we added a supplementary figure with pictures and quantifications of antero-posterior (Sup. Figure 1 C) and dorso-ventral length (Sup. Figure 1 D) of the analysed control and *vash1* morpholino injected embryos‘ development at 24, 34, 52 and 4dpf which shows no significant developmental delay and morphological defect. We added

© 2021. Published by The Company of Biologists under the terms of the Creative Commons Attribution License (https://creativecommons.org/licenses/by/4.0/).

a sentence in the Methods section (pages 11) to clarify the morphant's morphology and dosage-response curves.
We observe a 1-2 hour developmental delay of both the control and the *vash1* KD embryos compared to uninjected wild-type embryos, which led us to chose the 52 hpf time point to investigate the PLs. In uninjected embryos they are usually developed by 48hpf (Hogan et al., 2009).

With this, we believe we validated and strengthened the confidence in the results obtained with vash1 morpholino 1.

Minor comments:

a. The authors should back their qPCR data for *vash1* expression (Figure 1) by standard mRNA in situ hybridization, given the large degree of variability in *vash1* expression. Do they observe a dynamic expression in the vasculature using this technique?

We agree with the reviewer that an in situ hybridization would be beneficial to understand the expression pattern of *vash1* in wild type embryos. Accordingly, we performed an *in situ* hybridization for *vash1* mRNA that revealed expression at 24 and 34hpf in the dorsal aorta and perivascular tissue (Figure 1 E-G).

b. The number of nuclei per sprout in Fig. 3J does not correspond with the number of divisions per sprout presented in Fig. 3K. The authors observe one or two cell divisions per sprout in ctr MO injected embryos (Fig. 3K), however, Fig. 3J shows that the majority of ctr. sprouts contains only one cell. This is even more dramatic for *vash1* MO injected embryos, which can have up to four divisions, therefore should contain six cells. However, the maximum number of cells the authors report is three to four cells. How do these observations go together?

We believe these quantifications are not contradicting. The number of endothelial nuclei was assessed just prior to the connection to the ISV and the cell division quantification was done in a time-lapse from the time of secondary sprout emergence until the resolution of the 3-way connection. It is expected that there are more cell divisions during a longer time frame, as cells migrate dorsally or ventrally out of the sprout.

c. Fig. 5I and J have the same data points for control MO and *vash1* MO1. Does this mean that both graphs are from the same experiment? If so, the authors could combine the two graphs into one. If the two graphs are not from the same experiment, both would need to have independent controls.

Fig 5I and J (now fig. 6C and sup. fig. 2D) were from the same experiment. Since we now increased the replicates in both these experiments, they have independent control embryos and are consequentely presented in two different graphs (Fig. 6C and Sup. figure 2D).

d. The percentage of somites with PLs in *vash1* MO1 injected embryos in Fig. 5I is half the value shown in Fig. 5C. Although this kind of variability might be expected in biological samples, perhaps the authors could briefly discuss the issue and its implications on reproducibility in the manuscript so as to have the readers be aware of it, especially since the rescue of the *vash1* morpholino phenotype back to 50% from 25% is the same value the authors observed in the *vash1* KD alone in Fig. 5C. Here the value is 50% for the morpholino injection.

We added a sentence discussing the phenotypic variability in the discussion (see page 20), and we added a dosage response curves for the % of PLs (Sup. Figure 1 G-I), showing that embryos injected with the same amount of morpholino1, 2 and 3 against vash1 show variability in the percentage of somites with PLs at 52hpf.
We also noticed that the picture for the morphant on Fig. 5C had less PLs than the average in the quantifications (25% of 7 somites is 1,75 PLs) . We therefore added a more representative picture of PLs for *vash1* morphant in Fig. 5C, now Fig. 6C (about 1-2 somites/embryo exhibit PLs).

© 2021. Published by The Company of Biologists under the terms of the Creative Commons Attribution License (https://creativecommons.org/licenses/by/4.0/).

e. The Y-axis label is missing in Fig. 2H and Fig. 4J. Figure 5D lacks bars showing median and standard deviation.

Y-axis of Fig. 2H and 4J (now fig. 5J) correspond to ratios. We added AU/AU (arbitrary unit) to these graphs to make it clearer. We added the bars in Fig. 5D (now fig. 6D).

f. It would help to have an inference or conclusion at the end of each results section.

We appreciate this comment and added one conclusion sentence per results section (see pages 13-17).

Reviewer #3 (Significance (Required)):
Conceptual: As per my knowledge, this is the first study that looks at microtubule modifications in the context of a vertebrate organism past the gastrulation stage, as opposed to similar studies that have been done in cell culture or invertebrates (S. cerevisiae, C. elegans and D. melanogaster). Moreover, this study is one of few that address a novel link between the cytoskeleton and the process of cell fate specification.

Previous studies have separately shown that Vash1 limits angiogenesis and detyrosinates MTs. The current study combines the two observations in the context of endothelial cells, and hypothesizes that perhaps the function of Vash1 in limiting angiogenesis and at the same time promoting lymphatic development could be due to its role in MT modification at the molecular level and the consequent effect of this on cell division and/or fate specification at the cellular level. In short, this study aims to connect the long-standing gap in knowledge between cytoskeletal modifications and cell dynamics (in particular, division and specification) in a vertebrate organism. I therefore believe that the current study would be an exciting finding for research communities that study cytoskeletal influence on cellular dynamics and also those in the broad area of vascular biology.

My field of expertise relates to vascular biology, specifically developmental angiogenesis and the behavior of endothelial cells in zebrafish.

References

Barisic, M., Silva E Sousa, R., Tripathy, S. K., Magiera, M. M., Zaytsev, A. V., Pereira, A. L., Janke, C., Grishchuk, E. L., & Maiato, H. (2015). Microtubule detyrosination guides chromosomes during mitosis. *Science*, *348*(6236), 799-803. https://doi.org/10.1126/science.aaa5175

Hogan, B. M., Bos, F. L., Bussmann, J., Witte, M., Chi, N. C., Duckers, H. J., & Schulte-Merker, S. (2009). Ccbe1 is required for embryonic lymphangiogenesis and venous sprouting. *Nature Genetics*, *41*(4), 396-398. https://doi.org/10.1038/ng.321

Isogai, S, Horiguchi, M., & Weinstein, B. M. (2001). The vascular anatomy of the developing zebrafish: an atlas of embryonic and early larval development. *Developmental Biology*, *230*(2), 278-301. https://doi.org/10.1006/dbio.2000.9995

Isogai, Sumio, Lawson, N. D., Torrealday, S., Horiguchi, M., & Weinstein, B. M. (2003). Angiogenic network formation in the developing vertebrate trunk. *Development*, *130*(21), 5281-5290. https://doi.org/10.1242/dev.00733

Kimura, H., Miyashita, H., Suzuki, Y., Kobayashi, M., Watanabe, K., Sonoda, H., Ohta, H., Fujiwara, T., Shimosegawa, T., & Sato, Y. (2009). Distinctive localization and opposed roles of vasohibin-1 and vasohibin-2 in the regulation of angiogenesis. *Blood*, *113*(19), 4810-4818. https://doi.org/10.1182/blood-2008-07-170316

Koltowska, K., Lagendijk, A. K., Pichol-Thievend, C., Fischer, J. C., Francois, M., Ober, E. A., Yap, A. S., & Hogan, B. M. (2015). Vegfc Regulates Bipotential Precursor Division and Prox1

© 2021. Published by The Company of Biologists under the terms of the Creative Commons Attribution License (https://creativecommons.org/licenses/by/4.0/).

Expression to Promote Lymphatic Identity in Zebrafish. *Cell Reports*, *13*(9), 1828-1841. https://doi.org/10.1016/j.celrep.2015.10.055

Liao, S., Rajendraprasad, G., Wang, N., Eibes, S., Gao, J., Yu, H., Wu, G., Tu, X., Huang, H., Barisic, M., & Xu, C. (2019). Molecular basis of vasohibins-mediated detyrosination and its impact on spindle function and mitosis. *Cell Research*, *June*. https://doi.org/10.1038/s41422-019-0187-y

Lim, A. H., Suli, A., Yaniv, K., Weinstein, B., Li, D. Y., & Chien, C. Bin. (2011). Motoneurons are essential for vascular pathfinding. *Development*, *138*(21), 4813. https://doi.org/10.1242/dev.075044

Nicenboim, J., Malkinson, G., Lupo, T., Asaf, L., Sela, Y., Mayseless, O., Gibbs-Bar, L., Senderovich, N., Hashimshony, T., Shin, M., Jerafi-Vider, A., Avraham-Davidi, I., Krupalnik, V., Hofi, R., Almog, G., Astin, J. W., Golani, O., Ben-Dor, S., Crosier, P. S., … Yaniv, K. (2015). Lymphatic vessels arise from specialized angioblasts within a venous niche. *Nature*, *522*(7554), 56-61. https://doi.org/10.1038/nature14425

Nieuwenhuis, J., Adamopoulos, A., Bleijerveld, O. B., Mazouzi, A., Stickel, E., Celie, P., Altelaar, M., Knipscheer, P., Perrakis, A., Blomen, V. A., & Brummelkamp, T. R. (2017). Vasohibins encode tubulin detyrosinating activity. *Science*, *358*(6369), 1453-1456. https://doi.org/10.1126/science.aao5676

Second decision letter

MS ID#: DEVELOP/2020/194993

MS TITLE: Vasohibin-1 selectively regulates secondary sprouting and lymphangiogenesis in the zebrafish trunk

AUTHORS: Marta Bastos de Oliveira, Katja Meier, Simone Jung, Eireen Bartels-Klein, Baptiste Coxam, Ilse Geudens, Anna Szymborska-Mell, Renae Skoczylas, Ines Fechner, Katarzyna Koltowska, and Holger Gerhardt
ARTICLE TYPE: Research Article

I am happy to tell you that the referees are happy with your revisions and your manuscript has been accepted for publication in Development, pending our standard ethics checks. The referee reports on this version are appended below.

Reviewer 1

Advance summary and potential significance to field

This study investigated novel mechanistic details of lymphangiogenesis in zebrafish. It provides valuable new insights by analysing the function of the tubulin detyrosinase Vash1. While microtubule formation and spindle orientation are of importance to endothelial biology, this report provides the first evidence that tubulin detyrosination may be of importance for these processes. It therefore highlights a new cell biological aspect in zebrafish lymphangiogenesis.

Comments for the author

The authors have addressed the comments to their previous version carefully and satisfactorily, including a commendable rephrasing of the title. No further requests.

© 2021. Published by The Company of Biologists under the terms of the Creative Commons Attribution License (https://creativecommons.org/licenses/by/4.0/).

Reviewer 2

Advance summary and potential significance to field

The findings provide evidence for a role of vasohibin in regulating lymphatic blood vessel sprouting and suggest that this might be due to a function of vasohibin in regulating microtubule tyrosination. This work deepens our understanding of the role of a so far understudied mechanism controlling lymphatic cell specification and proliferation.

Comments for the author

The authors have addressed most of my concerns and significantly improved the manuscript.

© 2021. Published by The Company of Biologists under the terms of the Creative Commons Attribution License (https://creativecommons.org/licenses/by/4.0/).
